# DUET‐seq: An Open‐Source Droplet Platform for High‐Fidelity Joint Chromatin and Transcriptome Profiling Reveals Temporal Regulatory Decoupling in Single Cells

**DOI:** 10.1002/advs.77986

**Published:** 2026-09-27

**Authors:** Dong Cheng, Zijun Meng, Lan Wei, Pingjing Yang, Fanfan Zhang, Zhiyi Luo, Ting Lai, Chunli Li, Mengyao Zhao, Mengqin Xu, Jiaqi Wang, Linjun Li, Huarong Chen, Jin Li, Ailong Huang, Youquan Bu, Liuyang Zhao

**Affiliations:** ^1^ Department of Infectious Diseases Key Laboratory of Molecular Biology For Infectious Diseases (Ministry of Education) Institute For Viral Hepatitis The Second Affiliated Hospital Chongqing Medical University Chongqing China; ^2^ Department of Thoracic Surgery Department of Biochemistry and Molecular Biology College of Basic Medical Sciences The First Affiliated Hospital of Chongqing Medical University Chongqing Medical University Chongqing China; ^3^ Chongqing Key Laboratory of Translational Medical Research in Cognitive Development and Learning and Memory Disorders National Clinical Research Center for Child Health and Disorders China International Science and Technology Cooperation Base of Child Development and Critical Disorders Ministry of Education Key Laboratory of Child Development and Disorders Children's Hospital of Chongqing Medical University Chongqing China; ^4^ Chongqing Blood Center Chongqing China; ^5^ Department of Hepatobiliary Surgery The Second Affiliated Hospital of Chongqing Medical University Chongqing China; ^6^ Department of Cardiothoracic Surgery The First Affiliated Hospital of Chongqing Medical University Chongqing China; ^7^ Department of Anaesthesia and Intensive Care and Peter Hung Pain Research Institute The Chinese University of Hong Kong Hong Kong China; ^8^ Department of Laboratory Medicine The Affiliated Dazu's Hospital of Chongqing Medical University Chongqing China; ^9^ Molecular Medicine and Cancer Research Center Chongqing Medical University Chongqing China; ^10^ Reproductive Medicine Center the First Affiliated Hospital of Chongqing Medical University Chongqing China; ^11^ Key Laboratory of Laboratory Medical Diagnostics Chinese Ministry of Education Chongqing Medical University Chongqing China

**Keywords:** biology, chromatin, computational biology, gene expression, rna, transcriptome

## Abstract

Joint profiling of chromatin accessibility and gene expression in the same cell enables direct linkage of regulatory elements to transcriptional output, but existing approaches remain limited by low co‐capture sensitivity, proprietary platforms, and high costs. Here we present DUET‐seq, an open‐source droplet microfluidic platform for joint profiling of chromatin accessibility and gene expression from the same nucleus. DUET‐seq combines programmable dissolvable dual‐linker hydrogel beads with one‐step intra‐droplet RT‐PCR to physically co‐index RNA and transposed chromatin fragments, completing library preparation within 12 h. Optimization of joint reaction conditions, including suppression of residual Tn5 activity, achieves ∼3,200 genes per HEK293T cell while maintaining high‐quality chromatin accessibility profiles. By providing a fully disclosed reagent‐and‐device ecosystem, DUET‐seq enables researchers to optimize lysis and reaction chemistry for non‐standard tissues without proprietary constraints. We demonstrate the platform's versatility by mapping over 14,000 cis‐regulatory element‐to‐gene linkages in adult mouse brain and by profiling ∼29,000 nuclei across six stages of postnatal spermatogenesis, where paired measurements reveal two modes of temporal regulatory decoupling—epigenetic priming and chromatin inertia—that are inaccessible to unimodal assays. DUET‐seq thus provides an accessible, cost‐effective framework for joint single‐nucleus multi‐omic profiling, with broad applicability across developmental biology, disease epigenomics, and functional genomics.

## Graphical abstract

DUET‐seq is an open‐source droplet platform that jointly profiles chromatin accessibility and gene expression from the same nucleus. Dissolvable dual‐linker hydrogel beads and a one‐step intra‐droplet RT‐PCR co‐index both modalities in under 12 h at roughly $0.04 per cell. Across postnatal spermatogenesis, paired measurements expose two modes of temporal regulatory decoupling: epigenetic priming and chromatin inertia.

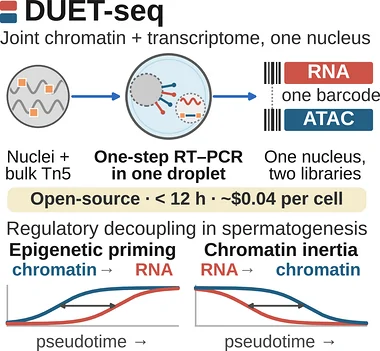

## 1 Introduction

A fundamental challenge in biology is to understand how gene expression programs are governed by cis‐regulatory elements in chromatin—yet the causal and temporal links between regulatory element activity and transcriptional output remain largely unresolved across cell types and developmental contexts [1, 2]. Elucidating these regulatory mechanisms necessitates the simultaneous profiling of the epigenome and transcriptome within the same single cell [3, 4]. Although unimodal single‐cell assays have transformed our view of cellular heterogeneity, each capture only one layer of the regulatory cascade [5]. Computationally integrating separate scRNA‐seq and scATAC‐seq datasets can approximate correspondence, but remains indirect and probabilistic, and it cannot physically link cis‐regulatory elements (CREs) to target genes—particularly in dynamic systems where chromatin and transcription are temporally offset [6, 7]. Joint profiling of the epigenome and transcriptome within the same single cell is therefore critical for resolving regulatory logic, yet current technologies impose significant barriers to widespread adoption [6].

A range of single‐cell multi‐omic technologies has been developed to address this need, yet three fundamental barriers limit their broad adoption. First, co‐capture biochemistry imposes sensitivity trade‐offs: combinatorial indexing approaches (e.g., sci‐CAR, SHARE‐seq) established feasibility but often yield limited molecular recovery per cell [8, 9, 10]. Droplet‐based methods (e.g., SNARE‐seq, ISSAAC‐seq, and SUM‐seq) improve throughput and automation via microfluidic compartmentalization [11, 12, 13, 14, 15, 16], yet joint chemistry frequently underperforms dedicated unimodal assays due to biochemical incompatibilities in co‐capture. Second, dependence on proprietary commercial ecosystems with undisclosed reagent formulations and device specifications prevents rational optimization for challenging tissues. Third, consumable costs remain prohibitive for longitudinal or large‐cohort studies [17]. Moreover, fixed bead architectures restrict assay customization and complicate the integration of additional molecular modalities. An open, high‐performance platform that addresses all three barriers would substantially accelerate the adoption of single‐cell multi‐omics.

Here we introduce DUET‐seq (**D**roplet‐based **U**nified **E**pigenome and **T**ranscriptome sequencing), an open‐source droplet microfluidic platform for high‐fidelity joint profiling of chromatin accessibility and gene expression in single nuclei. DUET‐seq integrates three key technical innovations to ensure flexibility, accessibility, and performance. (i) We synthesize the barcoded hydrogel beads in house rather than relying on proprietary beads: a dual‐linker acrydite primer carrying BC1 (n = 24) is incorporated during microfluidic polymerization, and two rounds of off‐chip split‐and‐pool synthesis append BC2 and BC3 (n = 384 each), so that 792 oligonucleotides yield 3.54 × 10^6^ distinct barcoded beads [18]. The dual‐linker design additionally allows the terminal capture sequences to be exchanged to support other assay configurations. (ii) We provide a fully “white‐box” ecosystem, including microfluidic designs and complete buffer formulations, enabling systematic optimization of parameters such as lysis and reaction chemistry for non‐standard tissues. (iii) We developed a streamlined one‐step RT‐PCR reaction that couples reverse transcription with amplification of transposed chromatin fragments within the same droplet, reducing total library preparation to <12 h while achieving high sensitivity and signal‐to‐noise at reduced cost.

We validate DUET‐seq through systematic benchmarking against current high‐throughput multi‐omic methods and through application to increasingly complex biological systems. In adult mouse brain, paired measurements enable same‐cell inference of cis‐regulatory element‐to‐gene associations without post hoc cross‐dataset integration. In a developmental times course of postnatal mouse spermatogenesis, DUET‐seq resolves two modes of temporal regulatory decoupling that are inaccessible to unimodal assays or computational integration: ‘epigenetic priming,’ in which chromatin opening precedes transcriptional induction during spermatogonial commitment, and ‘chromatin inertia,’ in which accessibility persists after transcriptional shutdown during spermiogenesis. Together, these results establish DUET‐seq as an open, cost‐effective, and high‐performance framework for joint single‐cell multi‐omic profiling.

## 2 Results

### 2.1 Design and Workflow of DUET‐seq

**FIGURE 1:**
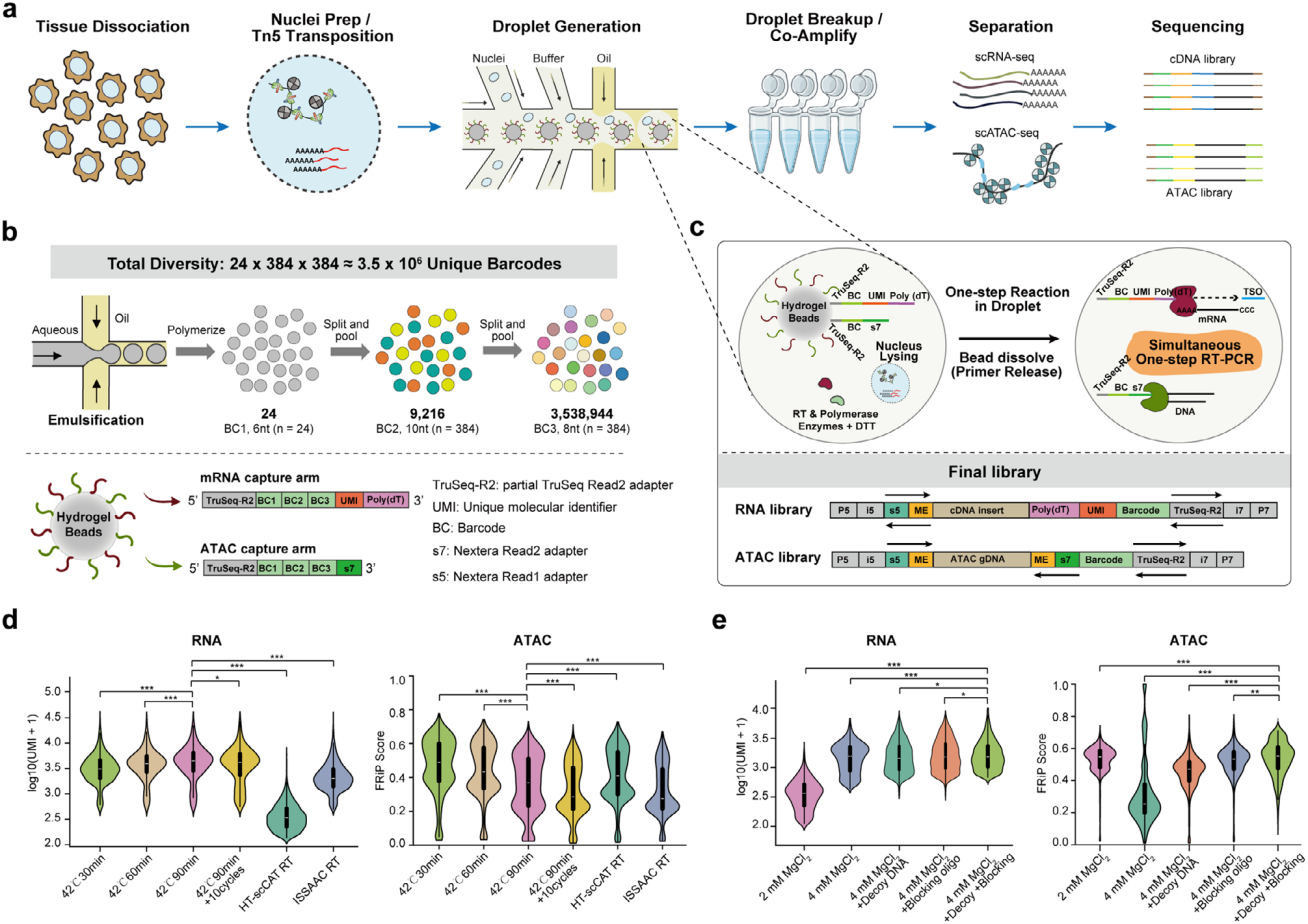
Design of the DUET‐seq platform and optimization of the one‐step intra‐droplet RT–PCR chemistry. (a) Schematic overview of the DUET‐seq workflow. Nuclei are transposed in bulk, co‐encapsulated with a dissolvable dual‐linker barcoded hydrogel bead, and processed within a single droplet reaction to jointly capture and index RNA and chromatin accessibility from the same nucleus. (b) In‐house bead synthesis and split‐and‐pool barcode assembly. All steps shown in this panel are performed during bead manufacture. An aqueous PEG/acrylamide solution containing an acrydite‐modified oligonucleotide (TruSeq‐R2 plus BC1) is emulsified on a microfluidic device and polymerized off‐chip into hydrogel beads; 24 independent polymerizations, each with a different BC1 (6 nt), are pooled. Two subsequent rounds of split‐and‐pool ligation append BC2 (10 nt, n = 384) and BC3 (8 nt, n = 384), so that 792 synthesized oligonucleotides generate 24 × 384 × 384 = 3,538,944 distinct barcodes while each finished bead carries a single, fully assembled 24‐nt barcode in ∼10^8^ copies. Bottom, the two linkers displayed on every bead share the same TruSeq‐R2 sequence and the same BC1–BC3 barcode and differ only in their 3′ module: a UMI followed by poly(dT) for mRNA capture, or an s7 adapter for priming transposed chromatin fragments. (c) Schematic mechanism of the key molecular reactions occurring within droplets. DTT‐mediated bead dissolution releases barcoded primers; cell lysis liberates nucleic acids; reverse transcription of RNA and amplification of transposed chromatin fragments occur within the same droplet. Element names follow Illumina adapter nomenclature: s5, Nextera Read 1 adapter sequence; s7, Nextera Read 2 adapter sequence; ME, mosaic end; TruSeq‐R2, Partial TruSeq Read 2 adapter. Full sequences are listed in Supplementary Table S1. (d) Violin plots showing RNA complexity (UMIs per nucleus; left) and ATAC quality (fraction of reads in peaks, FRiP; right) under six distinct RT–PCR thermocycling programs. Programs include a stepwise temperature ramp adapted from HT‐scCAT‐seq and a low‐temperature cycling protocol adapted from ISSAAC‐seq, as indicated in the legend. Center lines denote medians; boxes indicate interquartile ranges; whiskers extend to 1.5× IQR. Statistical significance was assessed by two‐sided Wilcoxon rank‐sum test comparing each program to 42°C 90 min; *p < 0.05, **p < 0.01, ***p < 0.001. (e) Optimization of the intra‐droplet RT–PCR chemistry in NIH/3T3 nuclei. All five conditions use the reverse‐transcription program selected in (d) and differ only as labelled: 2 mM or 4 mM MgCl_2_, and 4 mM MgCl_2_ plus 1 µM decoy DNA, 0.5 µM blocking oligo, or both. Left, RNA output; right, ATAC signal‐to‐noise (FRiP). Center lines denote medians; boxes indicate interquartile ranges; whiskers extend to 1.5× IQR. Statistical significance was assessed by two‐sided Wilcoxon rank‐sum test comparing each program to 4 mM MgCl_2_ with additives; *p < 0.05, **p < 0.01, ***p < 0.001. The graphical illustration in Figure 1a was created with BioRender (BioRender.com).

DUET‐seq enables concurrent measurement of chromatin accessibility and gene expression from the same nucleus at high throughput (Figure 1a; Figure S1; Table S1). The workflow begins with the isolation and permeabilization of nuclei, followed by bulk Tn5 transposition. These transposed nuclei are then co‐encapsulated in droplets with a single barcoded hydrogel bead and a unified RT‐PCR master mix (Figure S2a‐e). Distinct from existing single‐cell barcoding approaches, DUET‐seq uses a custom dual‐linker dissolvable hydrogel bead architecture (Figure 1b; Table S2). These barcode beads are prepared prior to the experiment and subsequently introduced during droplet generation. The beads are produced in‐house and preloaded with complete barcode sequences through a split‐and‐pool manufacturing process. Microfluidic droplet polymerization incorporates BC1 (6 nt, n = 24), and two rounds of off‐chip split‐and‐pool ligation append BC2 (10 nt, n = 384) and BC3 (8 nt, n = 384) [19]. The combinatorial assembly of these three barcode segments generates 3,538,944 unique barcode combinations. Split‐and‐pool is therefore used solely for barcode bead generation rather than sample indexing. The resulting barcode diversity provides sufficient capacity to minimize theoretical barcode collisions at the expected throughput. With the barcode architecture established, we next developed an in‐droplet strategy to couple chromatin accessibility and gene expression measurements. To physically link both modalities within droplets, we implemented a one‐step RT‐PCR strategy (Figure 1c). Upon droplet generation, DTT triggers bead dissolution, while detergent‐mediated lysis releases RNA and transposed chromatin fragments into a homogeneous reaction environment. The released poly(dT) primers initiate reverse transcription of poly(A)+ RNA, and the s7 capture linkers anneal to the newly synthesised s7 complement of the s7 adapter and prime pre‐amplification of the tagged fragments. As both products share the same cell barcode, the resulting RNA and ATAC libraries are intrinsically linked at the single‐cell level. After emulsion breaking, cDNA and ATAC amplicons are co‐amplified and then split for separate library construction, and the only indices added subsequently are the library‐level Illumina sample indices (Figure S2f). With library preparation completed in under 12 h and cost per sample substantially lower than comparable multi‐omic approaches (Table S3), the DUET‐seq workflow offers both efficiency and flexibility. The modular bead scaffold further allows straightforward adaptation by swapping terminal capture modules for additional readouts.

Because reverse transcription and amplification of transposition‐derived fragments must proceed in the same nanoliter droplet, we optimized intra‐droplet chemistry using a tiered strategy that distinguishes modality‐specific tuning from parameters that govern joint compatibility (Figure S3). Across a matrix of buffer components and cycling conditions—including MgCl_2_, NaCl, PEG8000, detergents, reducing agents and template‐switching oligos—we found that RT thermocycling and MgCl_2_ were major determinants of RNA recovery while shaping ATAC signal‐to‐noise (Figure 1d; Figure S3b). Notably, increasing MgCl_2_ or extending RT incubation improved RNA output but reduced ATAC quality, revealing a built‐in trade‐off that required a joint optimum (Figure 1d). We reasoned that this antagonism reflects a chemistry incompatibility: Mg^2^^+^ can reactivate residual Tn5 after encapsulation, leading to aberrant cleavage of transposed fragments and lowering ATAC signal‐to‐noise (Figure S3e), a constraint that likely contributes to the underperformance of joint multi‐omic workflows relative to dedicated single‐modality protocols [13, 20, 21]. To mitigate this, we adopted two complementary strategies originally developed by Zhang et al. [20]. to suppress residual transposase and free‐adapter activity in droplet workflows, and since applied in multiomic settings [13]: (1) introducing an unamplifiable DNA decoy to titrate excess Tn5 (“Decoy DNA”); and (2) adding a 3′‐blocked oligonucleotide complementary to the Tn5 adapter, which prevents free adapters from priming amplification (“Blocking oligo”). At 4 mM MgCl_2_, either additive alone restored FRiP to near the 2 mM level without reducing RNA output, and combining them performed at least as well as either alone (Figure 1e). We therefore adopted 4 mM MgCl_2_ with 1 µM Decoy DNA and 0.5 µM Blocking oligo as the final DUET‐seq chemistry (Figure 1e). Because both capture arms share a cell barcode, we further asked whether molecules of one modality could be mis‐assigned to the other. The two arms differ in the constant sequence immediately 3′ of the barcode: RNA molecules carry a UMI followed by poly(dT), and transposed fragments carry the s7 adapter. Every molecule therefore carries an intrinsic modality tag. Using this tag, chromatin‐derived reads accounted for 0.56% of the RNA libraries, and RNA‐derived reads for 0.38% of the ATAC libraries; the latter was independently supported by the fraction of ATAC fragments spanning annotated splice junctions (0.081%, compared with 0.059% for 10x Multiome). Both decoy DNA and the blocking oligonucleotide reduced these rates, and omission of reverse transcriptase or of transposition nearly abolished the corresponding library (Figure S3h‐j). Having finalized these reaction conditions, we performed species‐mixing experiments using human HEK293T and mouse NIH/3T3 cells to benchmark the platform. The species assignment results demonstrated high concordance between the two modalities, with an observed doublet rate of 9.1%, supporting robust droplet co‐encapsulation and barcode fidelity (Figure S2g).

### 2.2 Benchmarking DUET‐seq Against Existing Multi‐omic Platforms

**FIGURE 2:**
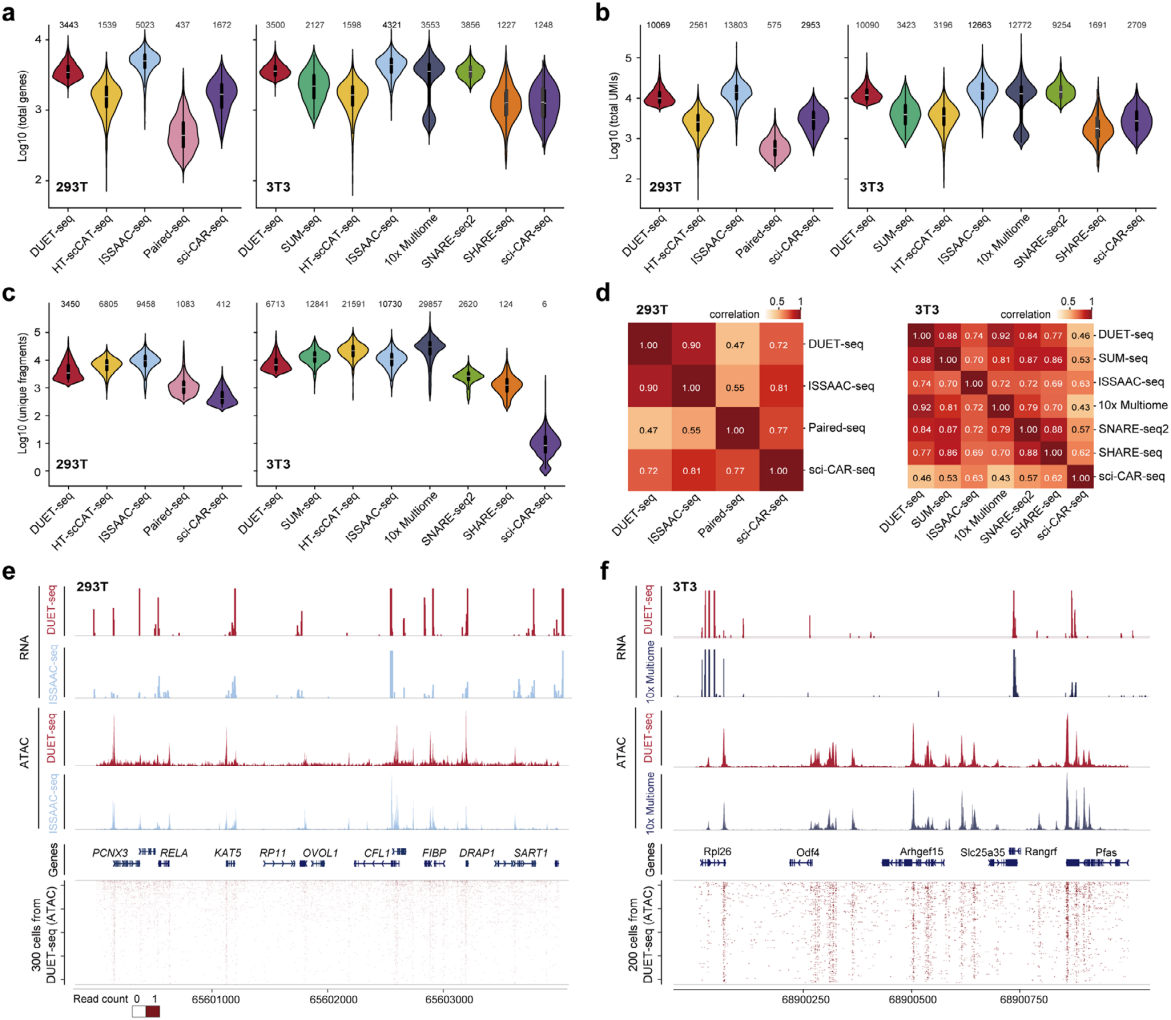
Validation and benchmarking of the DUET‐seq platform for simultaneous scRNA‐seq and scATAC‐seq. (a‐c) Violin plots comparing the performance of DUET‐seq against other methods. Metrics include (a) number of genes detected per cell, (b) number of UMIs per cell, and (c) number of unique ATAC fragments per cell. Boxplots within violins show the interquartile range with the median. The whiskers extend up to 1.5 times the interquartile range; the outliers are not displayed. Median numbers of detected genes are shown at the top of each box. (d) Heatmap showing Spearman correlation between transcriptome profiles generated by different approaches. All pairwise correlations were significant (two‐sided p < 0.001). Color intensity represents the Spearman correlation coefficient. (e) Representative genome browser tracks showing the aggregated signal of single cells (ATAC and RNA) of HEK293T cells from DUET‐seq and ISSAAC‐seq at a representative locus *chr11: 65,600,000‐66,000,000*. ATAC‐seq signals from 300 randomly selected single cells are shown at the bottom. (f) Representative genome browser tracks showing the aggregated signal of single cells (ATAC and RNA) of NIH/3T3 cells from DUET‐seq and 10x Multiome at a representative locus *chr11: 68,900,000‐69,000,000*. ATAC‐seq signals from 200 randomly selected single cells are shown at the bottom.

To rigorously evaluate DUET‐seq within the current technological landscape, we benchmarked its performance metrics against a broad spectrum of existing high‐throughput single cell multi‐omic methods. Comparative analysis across key performance metrics revealed that DUET‐seq achieves exceptional sensitivity, particularly in the transcriptomic modality (Table S4). For HEK293T cells, we detected a median of approximately 3,200 genes and 9,000 UMIs per cell, while NIH/3T3 cells yielded a median of 2,800 genes and 8,000 UMIs (Figure 2a,b). These values substantially exceed those typically reported for current droplet‐based approaches. In terms of chromatin accessibility, DUET‐seq yielded competitive data quality, with unique ATAC fragment counts exceeding those of sci‐CAR, Paired‐seq, SNARE‐seq2 and SHARE‐seq (Figure 2c). Although the unique fragment count was slightly lower than that of plate‐based or specific high‐depth methods like ISSAAC‐seq and 10x Multiome, DUET‐seq maintained high signal‐to‐noise ratio and characteristic nucleosomal periodicity, as evidenced by comparable Fraction of Reads in Peaks (FRiP) scores and strong TSS enrichment (Figure S2h,i). Global analysis revealed a high correlation between gene expression profiles from DUET‐seq, ISSAAC‐seq, SUM‐seq and 10x Multiome (Figure 2d). Visual inspection of the aggregated signals from single cells on a genome browser also demonstrated that DUET‐seq yields genomic profiles in strong concordance with data generated by both the 10x Multiome and ISSAAC‐seq platforms (Figure 2e,f). In HEK293T cells, DUET‐seq produced similar profiles compared to ISSAAC‐seq, a recently developed highly sensitive and flexible single‐cell multi‐omics method. In NIH/3T3 cells, DUET‐seq produced similar profiles compared to the 10x Multiome, a commercial multi‐omics method. Together, these results establish DUET‐seq as a high‐performance, flexible, and cost‐effective platform for joint single‐cell profiling that matches or exceeds the sensitivity of existing commercial and academic platforms (Table S4).

### 2.3 DUET‐seq Resolves Cellular Heterogeneity in Adult Mouse Brain and Enables Peak‐to‐gene Linking

**FIGURE 3:**
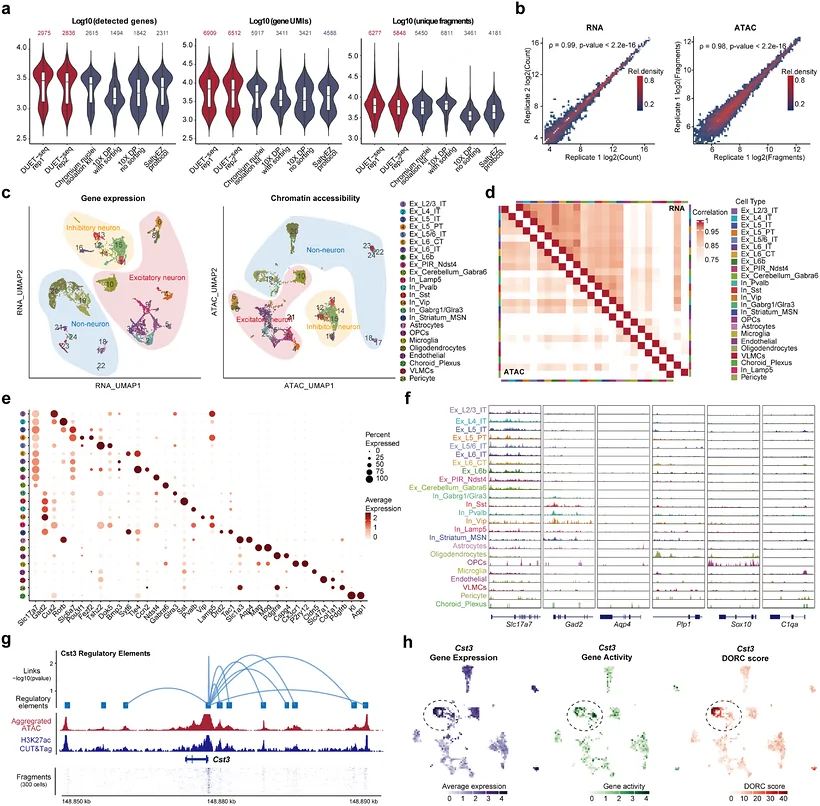
DUET‐seq resolves adult mouse brain cell types and enables peak‐to‐gene linking from paired measurements. (a) Violin plots comparing QC metrics (genes detected, UMIs, and unique ATAC fragments) between DUET‐seq and a public 10x Multiome brain dataset. Boxplots within violins show the interquartile range with the median. The whiskers extend up to 1.5 times the interquartile range; the outliers are not displayed. Median numbers of detected genes are shown at the top of each box. (b) Scatter plots demonstrating high reproducibility (Spearman's ρ) between two independent biological replicates for both RNA (left) and ATAC (right) modalities, and the corresponding two‐sided p‐value are shown on each panel. (c) UMAP projections of mouse whole brain cells, clustered and annotated based on RNA (left) and ATAC (right) profiles. (d) Spearman correlation coefficient between cell types in RNA (top right) and ATAC (bottom left) datasets. For RNA data, the correlation was calculated using the average gene expression per cell type, while the ATAC data correlation was calculated based on the average peak counts per cell type. All pairwise correlations were significant (two‐sided p < 0.001). Color intensity represents the Spearman correlation coefficient. (e) Dot plot illustrating the expression of canonical marker genes (RNA) across cell types. Dot size indicates the percentage of cells expressing the gene; color intensity represents the average expression level. (f) Genome browser tracks showing aggregated ATAC‐seq signal at cell‐type‐specific marker gene loci (gene activity) for major brain cell populations. (g) Representative example of peak‐to‐gene linkage analysis in mouse brain data. Loops denote the correlation of peak accessibility and RNA expression at the *Cst3* locus, loop height represents the significance of the correlation. Bulk H3K27ac CUT&Tag signal is shown for orthogonal validation. (h) Feature plots showing gene expression level (left), gene activity score (middle), and DORC score (right) for *Cst3*.

To test DUET‐seq in a highly heterogeneous tissue and to evaluate peak‐to‐gene inference from paired measurements, we profiled adult mouse brain in two biological replicates and retained 8,834 high‐quality nuclei after stringent filtering. Compared with a public 10x Genomics Multiome brain dataset processed with a matched analysis workflow, DUET‐seq recovered higher molecular complexity in both modalities (Table S5). For the RNA modality, DUET‐seq detected a median of 2,905 genes and 6,710 UMIs per nucleus, compared to 2,025 genes and 4,334 UMIs in the 10x Multiome dataset (Figure 3a). In the ATAC modality, DUET‐seq yielded a higher median number of unique nuclear fragments (6,500 vs. 5,900), while maintaining strong quality metrics, including Fraction of Reads in Peaks (FRiP) (Figure 3a; Figure S4a,b). Replicates were highly concordant for both RNA and ATAC pseudo‐bulk profiles (Spearman's ρ > 0.98), demonstrating robust technical reproducibility (Figure 3b).

Using the paired profiles, we resolved the major cell types of the adult mouse brain and linked accessible peaks to their target genes in the same nuclei. We performed unsupervised clustering based on RNA modality and transferred annotations to the matched ATAC profiles, resolving canonical neuronal and non‐neuronal populations of the brain [22, 23]. We identified 10 excitatory neuron subtypes (Ex, *Slc17a7*), 6 inhibitory neuron subtypes (In, *Gad2*), and 8 non‐neuron subtypes, including astrocytes (*Slc1a2*, *Slc1a3*), oligodendrocytes (*Plp1*), oligodendrocyte precursor cells (OPC, *Pdgfra*), microglia (*Hexb*), and endothelial cells (*Pecam1*) (Figure 3c–f; Figure S5). Marker‐gene expression patterns were mirrored by locus‐specific enrichment of chromatin accessibility (gene activity) at corresponding genes (Figure 3e,f), and lineage‐specific TF motif accessibility broadly agreed with TF expression (Figure S4c). Consistent with this modality concordance, subtype‐level pseudo‐bulk profiles showed high correspondence between RNA and ATAC (Figure 3d). To further benchmark annotation fidelity, we integrated DUET‐seq dataset with the 10x Multiome reference using Harmony and weighted nearest neighbor (WNN) analysis. Cells from both platforms intermingled extensively in the RNA manifold (Figure S4d‐f), supporting consistent biological structure across datasets. While the ATAC manifold showed modest platform‐associated variation, major cell classes and subtype relationships were preserved, indicating stable chromatin‐based resolution. Cell‐type proportions were broadly comparable between datasets, with minor differences likely reflecting sample preparation and nuclei isolation procedures in the public reference (Figure S4g).

Finally, we evaluated DUET‐seq for mapping cis‐regulatory interactions, a prerequisite for reconstructing gene regulatory networks [24]. Using paired RNA and chromatin accessibility profiles from the same nuclei, we identified thousands of peak‐to‐gene (P2G) linkages using FigR [25] (Figure S4h,i; Tables S6 and S7). This analysis systematically identified thousands of putative cis‐regulatory interactions, effectively bridging the gap between distal regulatory elements (such as enhancers) and their target genes across the genome. As a locus‐level example, we focused on *Cst3*, a gene previously reported to be highly enriched in microglia [26, 27]. We detected multiple distal regulatory peaks flanking the *Cst3* promoter that were significantly linked to *Cst3* expression (Figure 3g). These candidate elements overlapped bulk H3K27ac CUT&Tag signal, consistent with putative enhancer function. Consistently, UMAP projections showed that *Cst3* gene expression, gene activity, and the aggregated Domain of Regulatory Chromatin (DORC) score were enriched specifically in microglia (Figure 3h). Together, these results establish data quality and cis‐regulatory inference capability in a complex tissue, providing a benchmark for downstream developmental analyses.

### 2.4 Joint Multi‐omic Profiling of Mouse Testis Across Six Postnatal Stages

**FIGURE 4:**
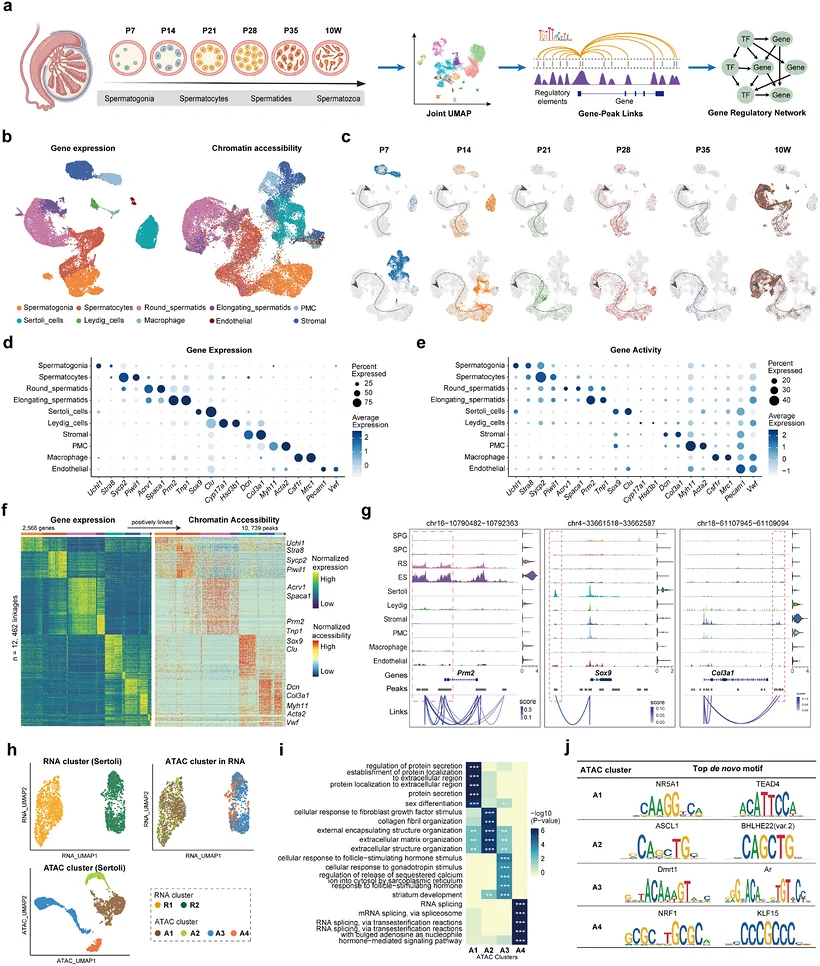
Joint single‐cell chromatin and transcriptome profiling of mouse testis development. (a) Schematic overview of mouse testis development across six key postnatal time points (P7, P14, P21, P28, P35, and 10 weeks). Nuclei were processed by DUET‐seq to jointly profile scRNA‐seq and scATAC‐seq from the same nucleus. (b) UMAP projections of the integrated testis dataset colored by cell type annotation. Projections are shown based on RNA modality (left) and ATAC modality (right). PMC denotes peritubular myoid cells. (c) Stacked bar plot visualizing the proportional changes in cell type composition across the six developmental time points. (d‐e) Dot plots showing marker gene expression (d) and inferred gene activity based on chromatin accessibility (e) across cell types (y‐axis). Dot size is proportional to the percentage of cells expressing the gene, and color intensity represents the average scaled expression or activity. (f) Heatmap shows normalized gene expression (left) and chromatin accessibility (right) for 12,462 significantly correlated peak‐gene pairs. Each row represents a putative positively associated peak–gene pair. The top annotation bar indicates the corresponding cell types involved in testis development. (g) Genome browser view of *Prm2*, *Sox9*, and *Col3a1* loci showing aggregated ATAC signal and peak‐to‐gene associations. Accessible peaks and linked peaks are shown for each gene. (h) UMAP visualization of RNA (top) and ATAC (bottom) profiles from 2,726 Sertoli cells (left). (i) Heatmap of aggregated ATAC signals across the top 200 cluster‐specific peaks for Sertoli ATAC subclusters (A1–A4). (j) The top de novo motifs enriched in the top 1,000 cluster‐specific peaks of Sertoli ATAC subclusters A1, A2, A3 and A4. Binomial tests were used to calculate the p‐value.

To demonstrate DUET‐seq's capacity for resolving temporal regulatory dynamics in a complex developmental system, we applied it to mouse testis across six postnatal stages: postnatal day (P) 7, P14, P21, P28, P35, and adulthood (10 weeks; 10 W) (Figure 4a). This strategy captures the complete continuum of spermatogenesis, from initial germ cell proliferation to sexual maturity [28]. After stringent quality control, 28,988 nuclei were retained for downstream analysis (Table S8). The resulting profiles exhibited robust data quality, including strong TSS enrichment, high fragment recovery, and clear nucleosomal periodicity (Figure S6a‐c). Interestingly, ATAC metrics—specifically unique fragment counts and FRiP scores—declined consistently from P7 to 10 W (Figure S6b). Reduced unique fragment counts are consistent with physiological chromatin condensation and ploidy reduction during spermatogenesis, which limits transposase accessibility in mature germ cells [29, 30]. The concurrent decline in FRiP, however, was primarily driven by post‐meiotic spermatids: depth‐controlled downsampling and cell‐state‐resolved analysis showed that FRiP was consistently lower in round and elongating spermatids relative to spermatogonia and spermatocytes within the same age (Figure S6g‐j), a pattern also reproduced in an independent mouse testis Multiome dataset [32]. In contrast, RNA complexity (genes and UMIs) remained stable across all time points (Figure S6a), providing a framework to resolve temporal decoupling between transcription and chromatin accessibility. Pseudo‐bulk correlations confirmed strong concordance among biological replicates (Figure S6d).

We embedded RNA and ATAC modalities using Seurat and Signac, respectively, and observed consistent overlap of biological replicates within each developmental stage. Cell types were annotated using RNA and labels were transferred to the paired ATAC profiles. The dataset resolved major germline and somatic populations based on canonical markers, including spermatogonia (SPG; *Uchl1*, *Stra8*), spermatocytes (SPC; *Sycp2*, *Piwil1*), round and elongating spermatids (SPT; *Spaca1*, *Prm2*), Sertoli cells (*Sox9*, *Clu*), Leydig cells (*Cyp17a1*), and macrophages (*Csf1r*) (Figure 4b–e; Figure S7a). Cells from different stages integrated well, and cell‐type composition shifted in line with developmental expectations (Figure S6e): P7 samples were enriched for somatic cells and early spermatogonia, whereas haploid spermatids emerged around P21 and became predominant by 10 weeks (Figure 4c; Figure S6f). We further calculated gene activity scores by summing unique chromatin fragments overlapping gene body and promoter regions. These RNA markers were mirrored by concordant chromatin accessibility patterns in corresponding ATAC clusters, indicating strong congruence between these two modalities (Figure 4e; Figure S7b).

Next, we leveraged the paired measurements to link cis‐regulatory elements (CREs) to gene expression. Differential expression analysis across cell types identified 5,067 DEGs (p‐value < 0.05 and |log2(fold change)| > 0.25). We then performed peak‐to‐gene linkage analysis by computing Pearson correlations between gene expression and accessibility of peaks within 500 kb of each transcription start site, consistent with prior large‐scale cis‐regulatory mapping studies [24, 31]. Positively correlated peak‐gene pairs were considered candidate enhancer‐gene associations (Tables S9 and S10). This yielded 12,462 significant links (correlation > 0, adjusted *p‐*value < 0.05), connecting 10,739 unique peaks to 2,566 genes (Figure 4f). Each linked gene was associated with a median of four peaks (min = 1, max = 28, mean = 4.185). These linkages recapitulated lineage‐restricted regulatory landscapes across germline and somatic compartments. For example, promoter‐proximal accessibility at the *Prm2* locus (chr16: 10,790,482–10,792,363) peaked in spermiogenic cells and coincided with maximal *Prm2* expression in round and elongating spermatids (Figure 4g). Multiple distal peaks linked to *Prm2* were selectively accessible in spermatids, consistent with activation of a post‐meiotic chromatin condensation program in which *Prm2* plays a central role [32]. In somatic lineages, peak–gene linkages at *Sox9* (chr4: 33,661,518–33,662,587) and *Col3a1* (chr18: 61,107,945–61,109,094) delineated Sertoli and stromal populations, respectively (Figure 4g), consistent with the established roles of *Sox9* in Sertoli identity and *Col3a1* as a stromal marker [33, 34].

To evaluate whether paired chromatin profiling can resolve regulatory heterogeneity beyond what transcription alone reveals, we examined Sertoli cells, which provide structural and metabolic support essential for germ cell development [35]. RNA‐based reclustering resolved two broad Sertoli states: an immature population enriched at P7 (R1) and a mature population spanning P14–10 W (R2) (Figure 4h; Figure S7e), consistent with the known developmental trajectory of Sertoli maturation [36, 37]. In contrast, the paired ATAC modality further stratified these states into four distinct chromatin subclusters (A1/A2 within R1 and A3/A4 within R2), supported by differential accessibility at cluster‐specific peaks (Figure S7c‐f). Motif enrichment linked these chromatin states to distinct regulatory programs (Figure 4i,j; Table S11): immature subclusters were enriched for motifs associated with sex differentiation (A1; NR5A1/TEAD4) [38, 39] and extracellular matrix organization (A2; ASCL1/BHLHE22) [40], whereas mature subclusters showed enrichment for androgen‐responsive regulation (A3; DMRT1/AR) [41, 42] and metabolic homeostasis (A4; NRF1/KLF15) [43, 44]. Together, these results indicate that Sertoli cells with similar transcriptomes can occupy distinct chromatin states, highlighting the ability of DUET‐seq to resolve regulatory heterogeneity and chromatin potential beyond transcriptional clustering alone.

### 2.5 Decoding Transcriptional and Epigenetic Programs During Spermatogonial Lineage Progression

**FIGURE 5:**
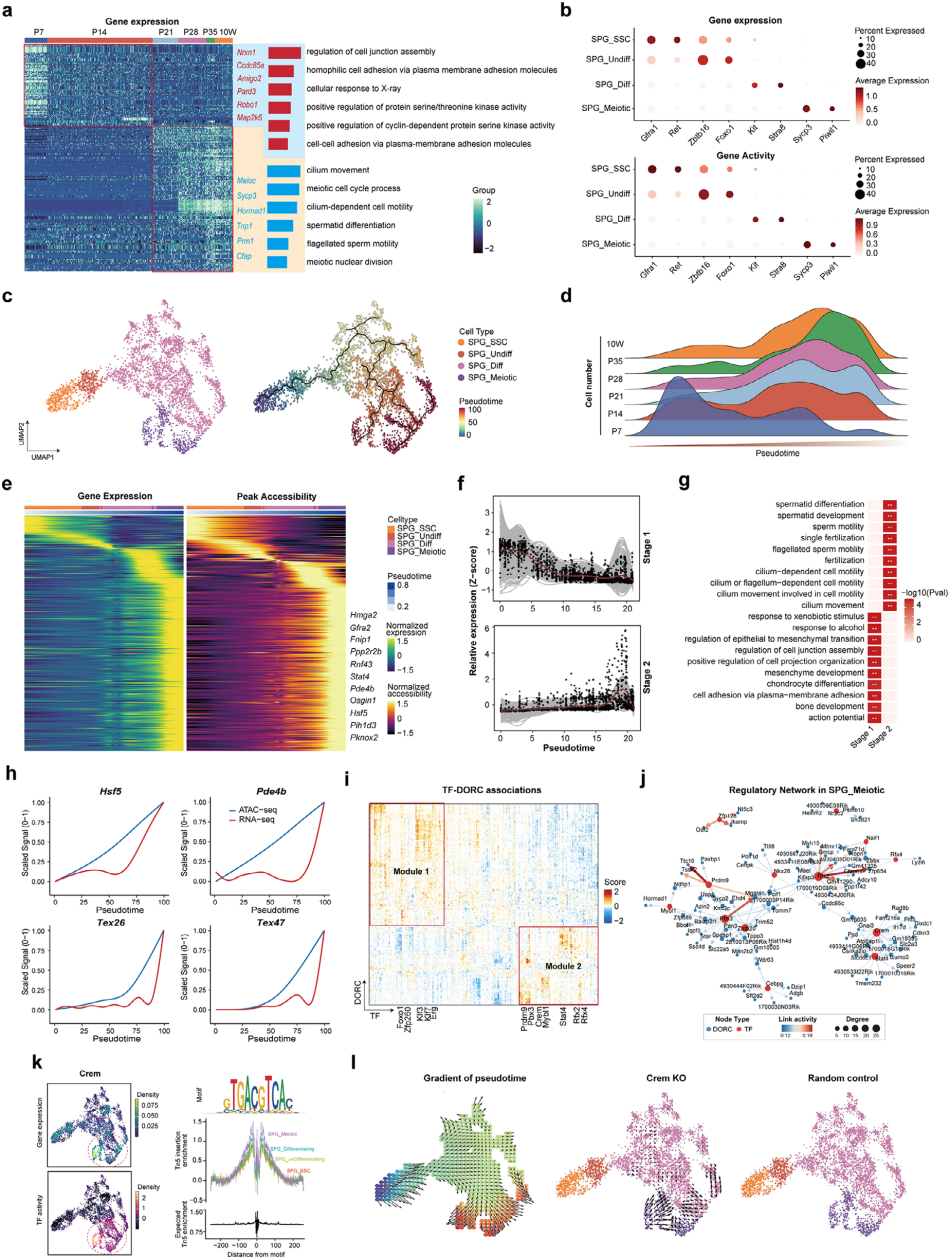
Epigenetic priming and regulatory network switching during spermatogonial lineage progression. (a) Heatmap of differentially expressed genes (DEGs) in spermatogonia (SPG) between early and late stages (left). Bar plot (right) shows Gene Ontology (GO) enrichment for terms related to self‐renewal (upregulated in early) and differentiation (upregulated in late). (b) Dot plots showing marker gene expression (top, RNA) and gene activity (bottom, ATAC) for defined SPG sub‐clusters. (c) UMAP visualization of SPG sub‐clusters (left, colored by cell type) and the inferred differentiation trajectory (right, colored by pseudotime). (d) Ridge plot showing the density distribution of spermatogonial cells along the inferred pseudotime trajectory, stratified by developmental time points (P7–10 W). (e) Heatmap showing gene expression (RNA) and chromatin accessibility (ATAC) along the pseudotime trajectory. Each row represents a putatively associated gene–peak pair. Top annotation bars indicate diffusion pseudotime and cell type. Columns are sorted by pseudotime. (f) Scatter plot with fitted lines shows the expression pattern for each group of lineage‐associated genes along the trajectory. The shaded areas around the fitted smooth line represent the error bands, indicating the 95% confidence interval. (g) Enriched GO terms for driver gene sets upregulated at stage 1 and stage 2 of the SPG trajectory. Dot size denotes enrichment ratio and color reflects significance. (h) Line plots of key differentiation genes showing that chromatin accessibility (ATAC, blue) changes are observed earlier than gene expression (RNA, red) along pseudotime. (i) Heatmap of TF‐DORC association scores calculated by FigR, highlighting candidate TFs (columns) predicted to be associated with specific DORCs (rows) during SPG differentiation. (j) Gene Regulatory Network visualization showing predicted associations between key TFs and target DORCs/genes, representing the inferred regulatory network during SPG differentiation. (k) UMAP projections of multimodal data for key TFs identified in (i), showing gene expression (RNA), TF activity inferred from motif accessibility, and motif accessibility (ATAC). (l) *In‐silico* perturbation of Crem using CellOracle. Vector‐field plots depict predicted changes in developmental trajectories upon TF knockout compared with control.

Continuous sperm production depends on the orderly transition of spermatogonial stem cells (SSCs) from self‐renewal to lineage specification [45, 46]. Although transcriptomic dynamics of spermatogonia (SPG) have been described, how chromatin states and upstream regulatory networks change across this transition remains less well resolved [47, 48]. To characterize the transcriptional transitions during SPG development, we first performed differential expression analysis across postnatal development (P7–10 W) within the SPG compartment. Based on temporal expression patterns and functional enrichment, SPG programs segregated into an early phase (P7–P14) and a late phase (P21–10 W) (Figure 5a; Figure S8a). Genes upregulated early were enriched for terms related to cell–substrate adhesion and signaling receptor activity (e.g., *Pard3*, *Robo1*), consistent with niche interactions required for stem cell anchoring [49] (Table S12). In contrast, late‐upregulated genes were enriched for spermatid differentiation and meiotic cell cycle programs (e.g., *Meioc*, *Sycp3*), indicating a shift toward lineage commitment [50] (Figure 5a). These divergent functionalities reflect a fundamental, programmatic transition of SPG from a stem cell–like, niche‐dependent state toward a lineage‐committed differentiation program.

To resolve heterogeneity within SPG population, we extracted the SPG profiles and re‐clustered them into four subtypes consistent with prior classifications [51]: Spermatogonial Stem Cells (SPG‐SSC; *Gfra1*, *Ret*), Undifferentiated SPG (SPG‐Undiff; *Zbtb16*, *Foxo1*), Differentiating SPG (SPG‐Diff; *Kit*, *Stra8*), and Meiotic‐entry cells (SPG‐Meiotic; *Sycp3*; *Piwil1*) (Figure 5b,c; Figure S8b). We observed a continuous progression from SSC to meiotic entry when SPG cells were projected into the low‐dimensional manifold (Figure 5c). Pseudotime trajectory reconstruction further confirmed a unified, unidirectional manifold, characterizing the continuous maturation from the SSC ground state toward meiotic commitment (SSC →Undiff →Diff →Meiotic) (Figure 5c). Notably, the inferred pseudotime trajectory closely recapitulated the actual postnatal ages of the cells: early‐stage SPG (P7–P14) localized at the beginning, while late‐stage SPG (P21–10 W) appeared toward the end (Figure 5d). This tight alignment confirms that SPG differentiation proceeds along a continuous and linear developmental axis, providing a robust foundation for dissecting the underlying regulatory logic.

We next quantified lineage‐associated transcriptional programs along pseudotime by correlating gene expression with trajectory progression. This analysis identified 3,714 lineage‐associated genes (correlation > 0.05 and adjusted *p‐*value < 0.05), including early markers (*Fst*, *Sema6a*), and late markers (*Dll1*, *Bmp3*) (Figure 5e; Table S13). To link these dynamics to cis‐regulatory changes, we performed peak–gene linkage analysis and identified 606 significant peak–gene pairs (correlation > 0 and adjusted *p*‐value < 0.05) (Figure 5e). Unsupervised clustering of linked genes separated two groups enriched at either the beginning or the end of the SPG trajectory (Figure 5f). Early‐upregulated linked genes were enriched for cell junction assembly and cell adhesion programs [52], whereas late‐upregulated linked genes were enriched for spermatid differentiation and sperm motility‐related terms, including flagellated sperm motility [53] (Figure 5g). These specialized functionalities characterize the progressive commitment toward the spermatid differentiation program. Notably, although RNA and ATAC showed broad concordance at a global level (Figure 5e), alignment along pseudotime revealed a consistent temporal offset between the two modalities. For representative genes, such as *Hsf5*, *Pde4b*, *Tex26*, and *Tex47*, accessibility at linked regulatory elements increased prior to detectable mRNA induction (Figure 5h). This “epigenetic priming” suggests that chromatin is reorganized in advance to establish a permissive regulatory state, poising differentiation‐associated genes for subsequent activation [54, 55, 56].

To uncover transcription factors governing spermatogonial self‐renewal and differentiation, we reconstructed gene regulatory networks (GRNs) using FigR (Figure S8f; Table S14). We identified 677 domains of regulatory chromatin (DORCs) based on significant peak–gene associations (*p*‐value < 0.05) within a ±50 kb window, integrating TF motif enrichment with chromatin accessibility. This analysis revealed 100 high‐confidence TFs (regulation score ≥ 1), which were organized into two distinct regulatory modules (Figure 5i; Figure S8c), as shown by heatmap visualization of TF‐DORC interactions based on chromatin accessibility patterns and associated TF motifs. Functional enrichment of the DORC genes indicated that Module 1 was primarily associated with self‐renewal functions, while Module 2 was enriched for differentiation‐related processes (Figure S8d). Module 1 contained TFs linked to stem cell maintenance and niche signaling, including Foxo1, Zfp260, Klf3, and Bcl6b, which help maintain the undifferentiated state. Conversely, Module 2 was enriched for TFs involved in meiotic entry and post‐meiotic programs, including Prdm9, Stat4, Rfx2, and Crem (Figure 5i).

To refine our analysis, we performed in silico perturbation of the FigR‐prioritized TFs to identify the core TFs regulating SPG self‐renewal and differentiation (Figure S8f; Table S15). Our perturbation analysis identified Erg as the most critical TF for self‐renewal. Erg regulates the self‐renewal of SSCs by controlling genes that maintain their undifferentiated state, thus preventing premature differentiation [57, 58]. Erg was found as a central node in the regulatory network (Figure S8g), likely regulating genes such as *Adgrl3*, *Akap1*, *Bicc1*, and *Ccnd3*, which may play roles in maintaining self‐renewal, though their exact functions require further investigation. TFs associated with SPG differentiation included Nr2c2, Crem, Atf1, Pbx3, and Rfx3 (Figure 5j). Crem, centrally located in this module, is pivotal for activating post‐meiotic transcriptional programs and supporting spermatogenic progression [59, 60] (Table S16). Crem, as an example, was predicted to regulate downstream targets such as *Izumo3*, *Cdkn3*, *Pde4b*, *Rad9b*, and *Dixdc1*, all of which are implicated in SPG differentiation (Figure 5j). These results underscore the critical involvement of specific transcription factors in regulating the transition from self‐renewal to differentiation in spermatogonia, providing a molecular framework for understanding SPG fate decisions.

To further prioritize candidate regulators of SPG differentiation, we focused on Crem, which showed enriched expression and chromatin‐associated regulatory activity in the SPG‐Meiotic population (Figure 5k). In silico loss‐of‐function simulation using CellOracle altered the predicted developmental vector field and reduced progression from undifferentiated toward meiotic states (Figure 5l), which confirmed its essential role in SPG differentiation. This computational perturbation supports CREM as a candidate regulator with a potentially strong influence on SPG differentiation, but does not by itself establish functional necessity. Together, our analysis provides insights into the core transcriptional regulators of SPG fate decisions, highlighting the importance of early epigenetic reconfiguration and subsequent TF‐driven network transitions in ensuring proper progression from self‐renewal to differentiation. The identification of critical TFs, such as Erg for self‐renewal and Crem for differentiation, offers valuable perspectives on the molecular regulation of spermatogenesis and potential targets for modulating SPG fate.

To examine whether the computationally nominated factors occupy the corresponding regulatory regions, we performed bulk CUT&Tag for CREM and RFX3 on mouse testis at P7, P21, P35, and 10 W (Figure S10). Both factors showed focal enrichment around ATAC peaks linked to spermatogonial lineage genes, with prominent signal at P7, and similar enrichment was observed at the representative *Pde4b* locus (Figure S10a,b). In the DUET‐seq developmental trajectory, these regulatory elements were accessible at P7 whereas the linked transcripts increased later, making the early CUT&Tag signal consistent with the proposed priming model. Because the CUT&Tag measurements were obtained from whole testis rather than purified spermatogonial populations, however, these data do not establish cell‐type‐specific temporal occupancy or causal regulatory function. One library was generated per factor per developmental stage without biological or technical replication.

### 2.6 Chromatin Inertia and Regulatory Logic Governing the Spermatid‐to‐sperm Transition

**FIGURE 6:**
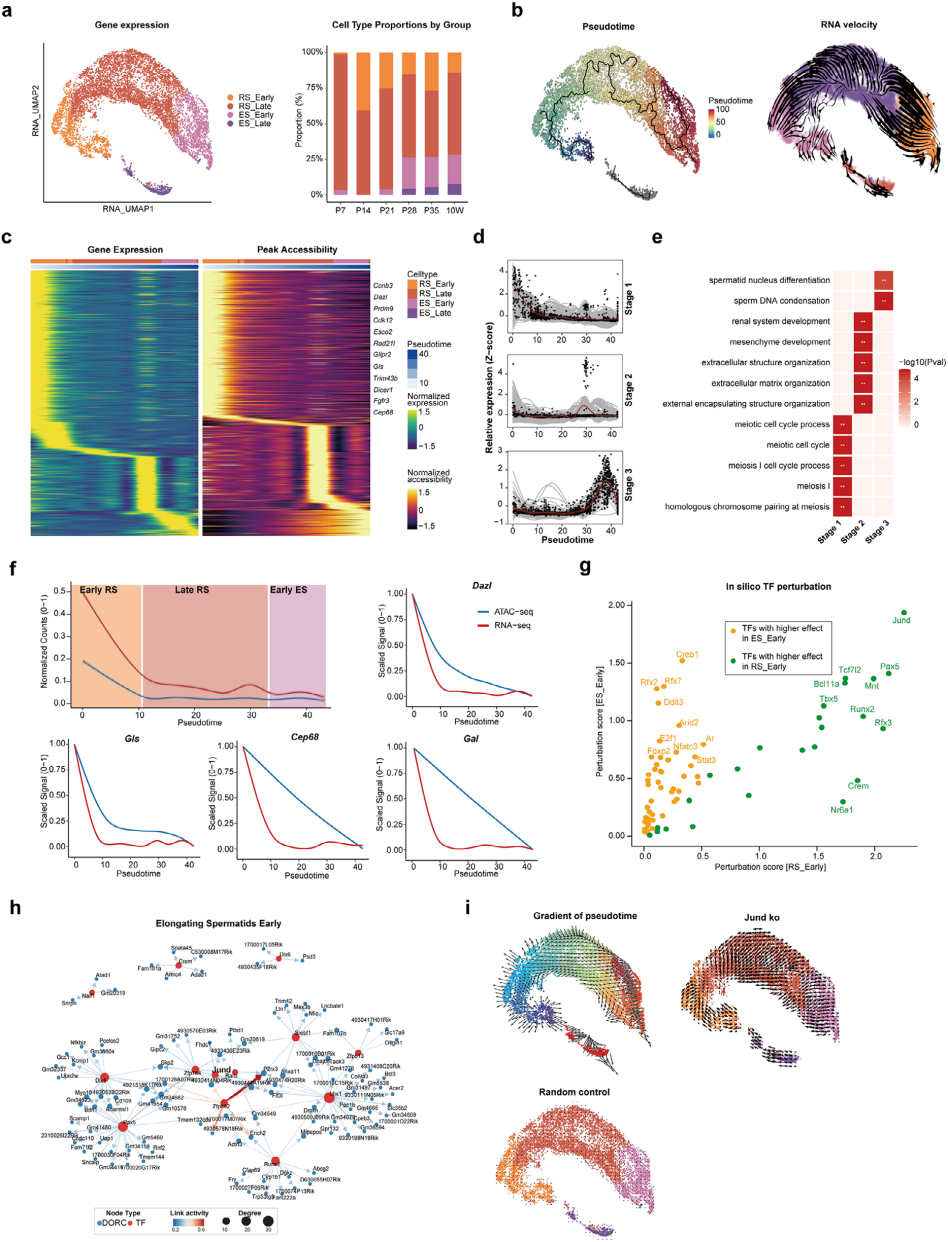
Chromatin accessibility dynamics accompany transcriptional silencing during spermiogenesis and highlight candidate regulatory factors. (a) UMAP visualization of post‐meiotic spermatid sub‐clusters colored by cell type (left). Bar plot (right) shows the cellular proportions of these subtypes across the six developmental time points. (b) Trajectory inference and transcriptional dynamics during spermiogenesis. Left: UMAP projection showing the inferred continuous pseudotime trajectory of spermatid differentiation, rooted at the early round spermatid stage. Right: RNA velocity streamplots showing the predicted direction of cell state transitions. (c) Dual‐modality heatmap of gene expression (RNA) and chromatin accessibility (ATAC) along the spermiogenesis pseudotime. The heatmap illustrates a temporal divergence between transcriptional downregulation and chromatin accessibility dynamics, a phenomenon referred to here as “chromatin inertia,” in which accessibility changes are observed to persist beyond transcriptional silencing. (d) Scatter plots with fitted smooth lines showing expression patterns of lineage‐associated gene groups along the trajectory. Shaded areas indicate 95% confidence intervals. (e) Enriched GO terms for gene sets upregulated at stage 1 and stage 2 of the spermiogenesis trajectory. Dot size denotes enrichment ratio and color reflects significance. (f) Representative examples illustrating a temporal offset between modalities, where chromatin accessibility at putatively associated peaks is observed beyond the corresponding mRNA expression along pseudotime. (g) Scatter plot of in silico TF perturbation scores, ranking transcription factors by their predicted influence on the RS_Early state (x‐axis) vs. the ES_Early state (y‐axis). Jund was ranked highest based on predicted influence scores. (h) Visualization of an inferred gene regulatory network connecting key TFs to predicted target genes, representing a putative regulatory framework associated with spermiogenesis. (i.) *In‐silico* perturbation of Jund using CellOracle. Vector‐field plots depict predicted changes in developmental trajectories upon TF knockout compared with control.

During spermiogenesis, the terminal phase of male germ cell maturation, the paternal genome undergoes extensive reorganization driven by the stepwise replacement of histones with protamines, leading to global chromatin condensation and progressive transcriptional shutdown [61, 62]. To resolve regulatory dynamics across this maturation continuum, we reconstructed 7,949 spermatids, in which we annotated four sequential states: early round (RS_Early), late round (RS_Late), early elongating (ES_Early), and late elongating (ES_Late) (Figure 6a; Figure S9a). Cell‐type proportions across developmental time points were consistent with a coordinated maturation wave, with elongating spermatids becoming predominant after P28 (Figure 6a). Trajectory inference captured a largely continuous progression from round to early elongating spermatids (RS_Early → RS_Late → ES_Early) (Figure 6b), whereas late elongating spermatids (ES_Late) appeared as a distinct, isolated population, consistent with extensive chromatin condensation and global transcriptional silencing at terminal maturation, which may reduce transcript‐based connectivity in trajectory inference. This framework enabled the dissection of transcriptional and chromatin dynamics during the final stages of spermatid differentiation.

To quantify transcriptional dynamics along this trajectory, we correlated gene expression with pseudotime and identified 943 lineage‐associated genes (correlation > 0.05 and adj. *p* < 0.05) (Table S17). Dynamic clustering separated these genes into three kinetic groups (Figure 6c,d). Group 1 decreased with maturation, group 2 remained relatively stable, and group 3 increased during terminal differentiation, enriched for spermatid nucleus differentiation and sperm DNA condensation [63] (Figure 6e). These late‐stage programs were most prominently induced in the late‐round to early‐elongating interval, consistent with the initiation of nuclear remodeling and chromatin condensation programs that accompany the onset of spermatid elongation (Figure 6c). To investigate the cis‐regulatory basis underlying these lineage‐associated transcriptional programs, we performed peak–gene linkage analysis and examined paired RNA and ATAC trajectories along spermiogenesis pseudotime. For representative genes such as *Dazl* (germ cell maintenance) [64], *Gls* (metabolism) [65], and *Cep68* (centrosome function) [66], transcript abundance declined across the round‐to‐elongating transition, whereas accessibility at linked regulatory loci persisted for a longer interval before decreasing (Figure 6f). We refer to this temporal relationship as chromatin inertia. Importantly, the magnitude and direction of the offset were heterogeneous across loci; we therefore interpret chromatin inertia as a gene‐set‐level tendency rather than a universal property of individual regulatory elements. Thus, the current data support a reproducible temporal decoupling between transcriptional decline and linked chromatin accessibility during spermiogenesis, while its molecular basis and locus‐specific causality remain to be established experimentally.

We next sought to identify trans‐acting regulators associated with progression along the spermiogenesis trajectory. Using FigR to integrate TF motif enrichment with DORC accessibility, we reconstructed a comprehensive TF‐DORC regulatory network comprising 823 DORCs and 129 high‐confidence TFs (regulation score ≥ 1) (Figure 6g; Figure S9b; Table S18). This global network analysis identified a cohort of high‐scoring regulators, including Runx2, Lhx1, and Jund (Figure S9c). Runx2 has been associated with transcriptional programs relevant to junctional and structural regulation in the testis [67]; and Lhx1 is a conserved regulator of urogenital lineage specification [68]. Collectively, these results nominate a set of TFs that may orchestrate chromatin and transcriptional programs underlying early post‐meiotic spermatid maturation.

To prioritize the most functionally influential TFs, we performed in silico perturbation using CellOracle across RS‐Early and ES‐Early (Figure 6g; Table S19). This analysis revealed stage‐specific regulatory hierarchies: Nr6a1 and Crem showed the highest perturbation scores in RS‐Early but low scores in ES‐Early, suggesting that they act as core regulators of the RS_Late. In contrast, Creb1, Rfx7, and Rfx2 exhibited the highest scores in ES‐Early, indicating central roles in early elongating spermatids. Jund was among the highest‐scoring TFs in both RS_Early and ES_Early, nominating it as a candidate regulator associated with the transition from round to elongating spermatids. Although Jund has been implicated in seminiferous epithelial homeostasis, its regulatory role during post‐meiotic spermatid maturation has remained largely unexplored [69, 70]. It exhibited enriched multimodal signals in round spermatids, implying that early post‐meiotic regulatory states influence subsequent morphological transitions (Figure 6g; Figure S9d). In silico loss‐of‐function simulation of Jund altered the predicted developmental vector field and reduced progression toward the elongating state (Figure 6g,i; Figure S9e; Table S20). These computational results prioritize Jund for future functional investigation but do not establish a causal or essential role in spermatid maturation.

## 3 Discussion

Joint single‐cell profiling of chromatin accessibility and gene expression provides a direct route to link cis‐regulatory state to transcription, yet widespread adoption has been limited by sensitivity trade‐offs, workflow complexity, and dependence on proprietary “black‐box” platforms. DUET‐seq addresses each of these barriers through an integrated design: programmable dual‐linker hydrogel beads, a fully transparent device‐and‐reagent ecosystem, and a streamlined in‐droplet RT–PCR workflow. This combination enables physical co‐barcoding of RNA and transposed chromatin fragments from the same nucleus, achieving high‐fidelity, high‐throughput paired profiles in a time‐ and cost‐efficient manner.

A central technical challenge in droplet‐based co‐capture is the biochemical antagonism between reverse transcription and transposition‐fragment amplification, where conditions favoring one modality often compromise the other [3, 71]. Through systematic optimization of intra‐droplet chemistry, we identified conditions that improve transcript recovery while maintaining robust chromatin profiles, and we further mitigated artifacts consistent with residual transposase activity by implementing complementary inhibition strategies. In addition, bead dissolution after encapsulation converts the droplet environment from a surface‐tethered capture scheme to a homogeneous reaction space, which likely increases effective molecular collision rates and supports efficient co‐barcoding. Together, these features provide a practical “white‐box” alternative to commercial systems, enabling laboratories to tune key parameters—such as lysis stringency and reaction composition—to accommodate challenging tissues and experimental constraints. DUET‐seq is not intended to displace existing joint assays on their own terms: ISSAAC‐seq attains its highest sensitivity in a plate‐based format with correspondingly limited throughput, and SUM‐seq attains very high throughput through combinatorial pre‐indexing that carries its own indexing infrastructure. DUET‐seq instead combines a fully disclosed device‐and‐reagent specification, an exchangeable dual‐linker bead architecture, 12 h library preparation at reduced cost, and the highest RNA sensitivity among the droplet‐based joint assays benchmarked here at matched depth (Table S4). Raw throughput per encapsulation run is the axis on which it does not compete.

Across applications, DUET‐seq demonstrated robust reproducibility and strong modality concordance. In adult mouse brain, paired measurements enabled large‐scale, direct mapping of cis‐regulatory elements to target genes without probabilistic integration, supporting large‐scale regulatory inference and the identification of cell‐type–specific regulatory modules. Applying DUET‐seq to postnatal mouse spermatogenesis revealed dynamic temporal relationships between chromatin accessibility and transcription that are difficult to resolve using unimodal assays [72]. Along the spermatogonial lineage, accessibility at linked regulatory elements often increased prior to induction of associated transcripts, consistent with early chromatin remodeling establishing permissive regulatory states—a phenomenon we term “epigenetic priming.” In contrast, during spermiogenesis, linked chromatin accessibility showed a gene‐set‐level tendency to decline later than transcript abundance. We refer to this temporal offset as “chromatin inertia.” The pattern is statistically supported and remains detectable after depth‐control analysis, but we regard it as hypothesis‐generating because direct single‐locus and factor‐level validation has not yet been performed. Beyond these temporal dynamics, paired profiles enabled reconstruction of regulatory networks along the developmental trajectory, revealing modules of transcription factors and cis‐regulatory elements that coordinate transitions from spermatogonial maintenance to meiotic and post‐meiotic programs. Perturbation simulations based on these networks nominated candidate drivers likely to influence germ‐cell progression, providing testable hypotheses for future functional validation. Collectively, these analyses demonstrate that DUET‐seq can link cis‐regulatory elements to target genes, resolve temporal regulatory logic, and identify candidate patterns of temporal decoupling and regulatory network architecture across complex tissues and developmental trajectories.

Beyond the germline, DUET‐seq also highlighted how chromatin‐based states can stratify cells that appear similar transcriptionally. In Sertoli cells, paired profiling revealed chromatin‐defined substates with distinct motif enrichments, consistent with the idea that chromatin potential can capture regulatory heterogeneity not fully reflected at the level of steady‐state RNA [10]. This feature may be particularly valuable in tissues where transcriptional profiles are constrained or partially decoupled from regulatory state, such as during differentiation transitions, stress responses, or late‐stage maturation processes. Importantly, this approach may be useful to investigate other developmental or disease contexts where transcriptional and chromatin states are partially decoupled, providing insights that are inaccessible to unimodal assays [73].

Several limitations provide directions for future improvements. First, as DUET‐seq operates on isolated nuclei, it primarily captures nuclear RNA; this may enrich for nascent or pre‐mRNA species and underrepresent stable cytoplasmic mRNAs, a consideration when interpreting steady‐state expression estimates [74]. Second, while paired measurements from the same cell substantially reduce ambiguity relative to post hoc dataset integration, peak‐to‐gene linkages remain correlative; establishing causality will require targeted perturbations such as CRISPRi/a screens at individual regulatory elements [75, 76]. CUT&Tag places CREM and RFX3 at the linked elements before the associated transcripts are induced, but occupancy is not function; establishing causality at these elements will still require targeted perturbation. Third, although depth‐controlled downsampling and cell‐state‐resolved analysis indicate that the FRiP decline in late‐stage spermatids reflects germ‐cell‐state‐associated chromatin remodeling rather than a uniform technical artifact (Figure S6g‐j), the correlative nature of pseudobulk RNA–ATAC alignment means that direct causal or single‐locus confirmation of chromatin inertia would benefit from further orthogonal validation, such as ATAC‐seq on FACS‐sorted spermatid populations matched by developmental stage. Fourth, although we provide open designs and reagent specifications, performance depends on careful control of bead synthesis and reaction conditions, motivating continued standardization and community‐driven benchmarking. Future extensions may include multi‐modal profiling—such as protein abundance or DNA methylation—within the same workflow, leveraging the modularity of DUET‐seq [77, 78]. Fifth, although the shared cell barcode is what couples the two modalities, it also means that modality assignment relies on the arm‐specific constant sequences rather than on independent barcodes; we quantified the resulting cross‐modality rate at 0.56% and 0.38% for the two directions and removed such reads during preprocessing, but residual mis‐assignment below this detection limit cannot be excluded. We note that contamination of either kind increases apparent concordance between modalities and therefore acts against, rather than in favour of, the temporal decoupling we report.

In summary, DUET‐seq provides an open, modular, and scalable platform for high‐fidelity paired chromatin accessibility and gene expression profiling that addresses three critical barriers to broad adoption of single‐cell multi‐omics. By lowering barriers imposed by proprietary systems and enabling direct observation of regulatory timing within the same cells, DUET‐seq offers a practical framework for building multi‐omic atlases and for dissecting how chromatin states can precede—or persist beyond—transcriptional changes during development and differentiation.

## 4 Methods

### 4.1 Microfluidic Droplet Generator Fabrication

Microfluidic droplet generators were fabricated using standard SU‐8 photolithography and PDMS soft lithography. Device designs were drafted in AutoCAD (R2014) and printed onto a chrome mask with a laser writer. To generate the master mold, SU‐8 3050 negative photoresist (Microchem) was spin‐coated onto a 4‐inch silicon wafer with a two‐step program: 500 rpm (100 rpm/s ramp) for 10 s to spread the resist, followed by 2000 rpm (300 rpm/s ramp) for 30 s to achieve a feature height of 70–80 µm. The wafer was soft‐baked, exposed to 365 nm UV through the chrome mask using a UV aligner (EVG‐620), and post‐exposure baked to complete cross‐linking. Unexposed resist was removed using SU‐8 developer, yielding the master mold.

For PDMS chip fabrication, PDMS monomer and curing agent (SYLGARD 184, Dow Corning) were mixed at a 10:1 ratio, degassed under vacuum, and poured over the SU‐8 master. PDMS was cured at 80°C for 4 h, peeled from the mold, and inlet ports were punched with a 1‐mm biopsy needle. The PDMS device and a clean glass slide were treated with high‐voltage oxygen plasma for 30 s and immediately bonded. To render channels hydrophobic, 5 µL of 2% Trichloro(1H,1H,2H,2H‐perfluorooctyl) silane in hydrofluoroether (HFE) was injected into each channel, incubated 10 min at room temperature, and removed with pressurized air. Assembled chips were baked at 100°C for 2 h prior to use.

### 4.2 Bead Synthesis and Split‐and‐pool Barcoding

All barcoded hydrogel beads used in this study were produced in‐house. Barcode assembly is completed entirely during bead manufacture, prior to any contact with biological material. Soluble beads were generated according to Hydrop with minor modifications [79, 80]. Briefly, 2 mL of monomer solution [6% acrylamide, 0.55% bisacryloylcystamine, TBSET buffer (10 mM Tris‐HCl pH 8.0, 137 mM NaCl, 2.7 mM KCl, 10 mM EDTA, 0.1% Triton X‐100), 12 µM acrydite‐modified oligonucleotide (TruSeq‐R2 + BC1, 6 nt), 0.6% ammonium persulfate] was emulsified into 50 µm droplets in HFE‐7500 oil containing EA‐008 surfactant. The acrydite‐modified oligonucleotide comprises  the partial TruSeq Read 2 sequence (TruSeq‐R2; 5′‐CAGACGTGTGCTCTTCCGATCT‐3′) followed by the first barcode segment BC1 (6 nt). Twenty‐four independent emulsification and polymerization reactions were performed in parallel, each with a distinct BC1 sequence, and the resulting bead batches were pooled after washing (n = 24). Polymerization, oil removal and washing were performed as described above.

For the first split‐and‐pool round, pooled beads were distributed evenly across a 384‐well plate. Each well received one distinct BC2 barcode primer (10 nt) pre‐annealed 1:1 with its splint (100 µM each) and phosphorylated with T4 PNK (37°C, 30 min; inactivated at 65°C, 20 min); ligation was performed with T4 DNA ligase (37°C, 1 h). Beads were then recovered from all 384 wells, pooled, washed, and redistributed across a second 384‐well plate for an identical round appending BC3 (8 nt, n = 384). In this second round, each well contained an equimolar mixture of two ligation oligonucleotides carrying the same BC3 but different 3′ modules, one ending in a 10‐nt UMI followed by poly(dT), the other ending in the 15‐nt s7 adapter. Both linker species on a given bead therefore carry an identical BC1–BC2–BC3 barcode, which is what allows RNA and ATAC molecules from the same nucleus to be matched.

The two rounds yield 24 × 384 × 384 = 3,538,944 barcode combinations from 792 synthesized oligonucleotides, while each individual bead displays a single 24‐nt barcode. Barcode sequences within each set were selected to have a minimum pairwise Hamming distance of 3, permitting unambiguous single‐mismatch correction against the whitelist. All barcodes, splint and linker sequences are provided in Table S2. To obtain single‐stranded barcodes, beads were incubated in denaturing buffer (100 mM NaOH, 0.5% Brij‐35) and neutralized.

### 4.3 Mice

C57BL/6J mice were housed under a 12 h light/dark cycle in a specific‐pathogen‐free facility with ad libitum access to food and water. All procedures were approved by the Experimental Animal Welfare Ethics Committee of Chongqing Medical University (approval number: IACUC‐CQMU‐2025‐06071) and conducted according to relevant ethical guidelines.

### 4.4 Cell Culture

HEK293T (human) and NIH/3T3 (mouse) cells were cultured in Dulbecco's Modified Eagle Medium (DMEM, Thermo Fisher) supplemented with 10% fetal bovine serum (FBS) and 1% penicillin–streptomycin. Cells were incubated at 37°C with 5% CO_2_. Adherent cells were dissociated using trypsin to generate single‐cell suspensions. To empirically determine the doublet rate of DUET‐seq, HEK293T and NIH/3T3 cells were mixed at a 1:1 ratio prior to nuclei isolation.

### 4.5 Mouse Brain

Whole brains were dissected from 8–9‐week‐old C57BL/6J mice and snap‐frozen in liquid nitrogen. Nuclei were isolated from frozen tissue using a modified HT‐scCAT‐seq protocol [11]. Briefly, tissue was thawed in 1 mL ice‐cold homogenization buffer (250 mM sucrose, 25 mM KCl, 5 mM MgCl_2_, 20 mM Tris‐HCl pH 8.0, 1 mM DTT, 1× Protease Inhibitor (Roche), 0.5 U/µL RiboLock RNase Inhibitor (Thermo Fisher), 1 U/µL Recombinant RNase Inhibitor (Takara), 1% BSA) in a 2 mL Dounce homogenizer. Homogenization was performed with 10 strokes of the loose pestle followed by 10 strokes of the tight pestle. The homogenate was filtered through a 40 µm strainer and purified via iodixanol density gradient centrifugation to remove myelin and debris, minimizing mitochondrial DNA contamination. Purified nuclei were resuspended in BSA‐containing buffer with RNase inhibitors and quantified using DAPI staining.

### 4.6 Cell Dissociation From Mouse Testis

Testes were collected from C57BL/6J mice at postnatal day 7 (P7), P14, P21, P28, P35, and 10 weeks (10 W) to profile testicular development. A two‐step enzymatic digestion was used to obtain single‐cell suspensions [81]. Briefly, the tunica albuginea was removed, and seminiferous tubules were incubated with 1 mg/mL collagenase IV (Sigma) in HBSS at 37°C to dissociate the tubules. After washing, tubules were further digested with 0.25% Trypsin‐EDTA (Thermo Fisher) and DNase I (Sigma) to release individual cells. Digestion was quenched with FBS‐containing medium, and the resulting cell suspension was filtered sequentially through 70and 40 µm strainers. Cells were pelleted, resuspended in 1× PBS with 0.04% BSA, and counted using an automated cell counter.

### 4.7 Nuclei Preparation

For cell line and testis samples, nuclei were isolated from single‐cell suspensions using a modified Omni‐ATAC protocol [82]. Cells were washed once with ice‐cold DPBS containing 0.5% BSA and 0.2 U/µL Murine RNase Inhibitor (Yeasen), and viability was assessed by Trypan blue staining (Thermo Fisher), requiring >90% cell viability. Approximately 5×10^5^ cells were resuspended in 100 µL of ice‐cold lysis buffer (10 mM Tris‐HCl pH 7.5, 10 mM NaCl, 3 mM MgCl_2_, 0.1% Tween‐20, 0.1% IGEPAL CA‐630, 0.01% Digitonin, 0.5 U/µL RiboLock RNase Inhibitor (Thermo Fisher), 1 U/µL Recombinant RNase Inhibitor (Takara), 1 U/µL Murine RNase Inhibitor (Yeasen)) and incubated on ice for 5 min. Lysis was stopped by adding 1 mL of wash buffer (10 mM Tris‐HCl pH 7.5, 10 mM NaCl, 3 mM MgCl_2_, 0.1% Tween‐20), and nuclei were pelleted at 500 × g for 5 min at 4°C. The nuclei pellet was resuspended in PBS with RNase inhibitors and quantified using DAPI staining.

### 4.8 Transposome Complex Assembly

Tn5 transposome complexes were freshly assembled on the day of each experiment following the Vazyme protocol. Oligonucleotides for ME_s5, ME_s7, and ME_bottom were dissolved to 10 µM in 0.1× TE buffer. To generate double‐stranded s5_adapter, 10 µL of ME_s5 (10 µM) was mixed with 10 µL of ME_bottom (10 µM), and the same procedure was applied to s7_adapter. The mixtures were annealed in a thermocycler using the following program: 98°C for 3 min, slow cooling to 16°C at 0.1°C/s, and hold at 16°C. For transposome assembly, 3.5 µL of each annealed adapter (5 µM) was combined, followed by the addition of 4 µL TruePrep Tagment Enzyme (500 ng/µL, Vazyme) and 39 µL coupling buffer to a final volume of 50 µL. The reaction was incubated at 30°C for 1 h with shaking at 350 rpm in a thermomixer. Assembled transposomes were used immediately or stored at −20°C for up to one month.

### 4.9 Chromatin Tagmentation

For each sample, approximately 100,000 isolated nuclei were used. The nuclei pellet was resuspended in 100 µL of freshly prepared chromatin tagmentation reaction mixture. The final reaction contained 1× THS TD buffer (prepared from 4× stock: 132 mM Tris‐HCl pH 8.0, 264 mM potassium acetate, 40 mM magnesium acetate, and 64% N,N‐dimethylformamide), 0.01% Digitonin, 0.5 U/µL RiboLock RNase Inhibitor (Thermo Fisher), 1 U/µL Recombinant RNase Inhibitor (Takara), 1 U/µL Murine RNase Inhibitor (Yeasen), 2.5 µL of the assembled Tn5 transposome complex, and nuclease‐free water to 100 µL. The reaction was incubated at 30°C for 30 min with 800 rpm shaking in a thermomixer. Tagmentation was stopped by adding 100 µL of 2x Stop Buffer (final concentration: 1× PBS, 20 mM EDTA, 2% BSA) and incubating on ice for 5 min. Nuclei were pelleted at 800 g for 5 min at 4°C, washed twice with 500 µL of ice‐cold 0.5x DPBS containing 0.5% BSA and 0.2 U/µL Murine RNase Inhibitor (Yeasen), and finally resuspended in 50 µL of 0.5x DPBS with 0.5% BSA for droplet generation.

### 4.10 Droplet Encapsulation, RT‐PCR, and Barcoding of DUET‐seq

Tagmented nuclei were loaded onto the DUET‐seq microfluidic chip at a limiting concentration (∼1,000 nuclei/µL) and co‐encapsulated into nanoliter droplets with single barcoded hydrogel beads. Each droplet contained one nucleus, one bead, and the one‐step RT‐PCR master mix. The final droplet reaction composition was optimized for both reverse transcription and PCR and included: 25 mM Tris‐HCl pH 8.3, 30 mM NaCl, 4 mM MgCl_2_, 0.20% IGEPAL CA‐630, 8 mM DTT, 1.75% PEG8000, 1 M Betaine, 3.5 mM dCTP, 0.5 mM dNTP mix, 1 µM TSO oligo, 1 µM Decoy DNA, 0.5 µM blocking oligo, 0.5 U/µL RiboLock RNase Inhibitor (Thermo Fisher), 1 U/µL Recombinant RNase Inhibitor (Takara), 1 U/µL Murine RNase Inhibitor (Yeasen), 4 U/µL Maxima H minus Reverse Transcriptase, and 0.03 U/µL KAPA HiFi HotStart DNA Polymerase.

Upon encapsulation, nuclei were lysed, releasing mRNA and tagmented DNA fragments. Within the droplets, mRNA molecules were captured by the bead‐attached poly(dT) primers and reverse‐transcribed into cDNA using a template‐switching oligo (TSO), while the released s7 capture linkers annealed to the gap‐filled complement of the s7 adapter of tagmented fragments and primed their extension and pre‐amplification. The collected emulsion was transferred to PCR tubes and thermocycled using the following program: 42°C for 90 min (reverse transcription), 72°C for 5 min (gap fill‐in), 95°C for 3 min (initial denaturation), and 10 cycles of PCR (98°C for 20 s, 60°C for 30 s, 72°C for 1 min) for initial ATAC amplification.

### 4.11 Post‐Droplet Processing and Co‐Amplification

Droplets were disrupted by adding 5 µL of 1H,1H,2H,2H‐perfluorooctanol (PFO), and the aqueous phase was recovered. RT–PCR products were purified using 1.8× VAHTS DNA Clean Beads (Vazyme) and eluted in 70 µL of 0.1× TE buffer. The entire eluate (67 µL) served as template for co‐amplification in a 100 µL reaction containing 1× KAPA HiFi Buffer, 0.3 mM dNTPs, 0.4 µM Forward Primer (TruSeq‐R2 primer), 0.2 µM Reverse Primer (TSO primer), 0.2 µM Reverse Primer (s5 primer), and 0.02 U/µL KAPA HiFi Polymerase. PCR cycling was performed as follows: 95°C for 3 min, 7 cycles of 98°C for 20 s, 60°C for 30 s, 72°C for 4 min, followed by a final extension at 72°C for 5 min. The amplified product was purified with 1.4× VAHTS DNA Clean Beads and eluted in 75 µL of nuclease‐free water, then split evenly into two 37.5 µL aliquots for separate ATAC and RNA library preparation.

### 4.12 Library Preparation

The 35.5 µL ATAC DNA template was subjected to final indexing PCR in a 50 µL reaction containing 1× KAPA HiFi Buffer, 0.3 mM dNTPs, 0.2 µM Forward Primer (Nextera i5 index primer), 0.2 µM Reverse Primer (TruSeq i7 index primer), and 0.02 U/µL KAPA HiFi Polymerase. PCR cycling was: 95°C for 3 min; 8–10 cycles of 98°C for 20 s, 60°C for 30 s, 72°C for 1 min; final extension at 72°C for 5 min. The library was purified via double‐sided size selection with VAHTS DNA Clean Beads (0.55× – 1.0×) and eluted in 23 µL of 0.1× TE buffer.

The 35.5 µL cDNA template was first enriched in a 50 µL PCR reaction containing 1× KAPA HiFi Buffer, 0.3 mM dNTPs, 0.2 µM Forward Primer (TruSeq‐R2 primer), 0.2 µM Reverse Primer (TSO primer), and 0.02 U/µL KAPA HiFi Polymerase. PCR cycling was: 95°C for 3 min; 6–8 cycles of 98°C for 20 s, 62°C for 30 s, 72°C for 4 min; final extension at 72°C for 5 min. The product was purified with 0.6× VAHTS DNA Clean Beads and eluted in 23 µL of 0.1× TE buffer. Approximately 5–6 ng of purified cDNA was tagmented in a 10 µL reaction containing 1× TD buffer (40 mM Tris‐HCl pH 7.5, 20 mM MgCl_2_, 20% DMF) and 0.4 µL Vazyme Tn5 transposome (Tn5 loaded with two identical Nextera Read 1 adapters, Tn5‐s5/s5) at 55°C for 10 min. The reaction was stopped with 2.5 µL of 0.2% SDS, and tagmented cDNA was subjected to final indexing PCR in 50 µL containing 1× KAPA HiFi Buffer, 0.3 mM dNTPs, 0.2 µM Forward Primer (Nextera i5 index primer), 0.2 µM Reverse Primer (TruSeq i7 index primer), 0.025% Tween‐20, and 0.02 U/µL KAPA HiFi Polymerase. Cycling: 72°C for 5 min (gap filling), 95°C for 3 min, 6 cycles of 98°C for 20 s, 65°C for 30 s, 72°C for 60 s; final extension at 72°C for 5 min. The RNA library was purified using VAHTS DNA Clean Beads (0.50×–0.75×) and eluted in 20 µL of 0.1× TE buffer.

### 4.13 Sequencing

Library concentration was determined with a Qubit fluorometer and size distribution assessed using an Agilent Bioanalyzer. ATAC and RNA libraries were pooled and sequenced on the Illumina or GeneMind platform. RNA libraries used a FCH 300‐cycle kit (Read 1: 150 cycles, Index 1: 8 cycles, Index 2: 8 cycles, Read 2: 150 cycles), while ATAC libraries used a 100‐cycle kit with custom sequencing primers (Read 1: 54 cycles, Index 1: 8 cycles, Index 2: 24 cycles, Read 2: 54 cycles).

### 4.14 Single‐cell Data Preprocessing

Prior to alignment, we performed cell barcode identification and correction for both scATAC‐seq and scRNA‐seq modalities. For the ATAC library, the Index 2 read returns the 24‐nt DUET‐seq cell barcode rather than the i5 sample index; samples are therefore demultiplexed by the i7 index. A custom script was utilized to match the raw barcode sequences (extracted from Index 2 for ATAC and Read 2 for RNA) against the DUET‐seq barcode whitelist. Barcodes with a Hamming distance ≤ 1 were corrected to the nearest whitelist sequence, ensuring high‐fidelity cell identification and consistent cross‐modal integration. For the scATAC‐seq component, the reformatted FASTQ files (Read 1, Read 2, and Index 2) were processed using Cell Ranger ATAC (v.2.1.0) from 10x Genomics. Fragment files and the peak‐by‐cell matrix were generated using the standard pipeline, with peak calling performed via the integrated MACS2 tool [83]. For the scRNA‐seq component, the reformatted Read 1 and Read 2 (containing the cell barcode and 10 bp UMI) were processed using Cell Ranger (v.8.0.0). The pipeline executed read alignment against the reference transcriptome, followed by UMI counting to generate the gene‐by‐cell expression matrix.

### 4.15 Assessment of Cross‐modality Contamination

Cross‐modality reads were identified from the constant sequence immediately 3′ of the parsed cell barcode. Reads whose following 10 nt matched GTCTCGTGGG (Hamming distance ≤ 1) were classified as transposition‐derived; reads matching the NNWNNNWNNN UMI pattern were classified as RNA‐derived. Classification was performed only for reads with a complete BC1–BC2–BC3 assignment, giving an analytical false‐positive rate of ≈4 × 10^−^^6^. Classified reads were counted per library and per cell barcode, and transposition‐derived reads were removed prior to Cell Ranger quantification. To assess the reciprocal direction, an aliquot of each ATAC library was re‐sequenced with the barcode read extended to 40 cycles, enabling the UMI and poly(dT) of RNA‐derived molecules to be read for barcodes of any length. ATAC reads were also realigned with STAR (v2.7.2) against the mm10 genome with the GENCODE annotation, and fragments containing an N operation in the CIGAR string that spanned an annotated exon–exon junction were counted. Matched analyses of 10x Multiome ATAC data (NIH/3T3 data from ISSAAC‐seq) were used as references.

### 4.16 Single‐cell Metric Comparison to Other Methods

We downloaded FastQ files of cell line data from other multi‐omics technologies. To ensure uniformity, we downsampled all data sets to 50,000 read pairs per cell for each modality. We then applied the ISSAAC‐seq preprocessing workflow to these data consistently. For scATAC‐seq data, we employed Chromap (v0.2.7) [84] for read mapping. Meanwhile, scRNA‐seq reads were analyzed using STARsolo (v2.7.11b) [85]. The resulting count matrices were used to generate the quality metrics. For cross‐platform correlation analysis, we generated pseudo‐bulk profiles by summing gene counts across all cells for each technology. Genes with zero total counts across all platforms were excluded to ensure numerical stability during Spearman correlation coefficient calculation. Furthermore, to evaluate the performance across various experimental conditions during methodological optimization, we standardized the sequencing depth to 10,000 read pairs per cell for each condition to ensure a fair comparison.

### 4.17 Species Mixing Experiment

For the species mixing experiment, the resulting data were mapped to a hg38 and mm10 combined reference genome using chromap (ATAC) and STARsolo (RNA). Cell barcodes with more than 80% of reads mapped exclusively to a single genome were classified as singlets; others were classified as doublets. Reads mapped to each genome in each cell barcode were counted. Of note, this doublet rate reflects the pre‐filtering observation; because homotypic doublets (same‐species pairs) cannot be distinguished from singlets in a barnyard assay, the measured rate represents a lower bound of the true doublet frequency.

### 4.18 Mouse Brain Data Analysis

The mouse brain multi‐omics data were analyzed using Seurat (v5.3.0) [86] and Signac (v1.14.0) [87]. For the RNA modality, we applied SCTransform to normalize gene expression while regressing out mitochondrial content. For the ATAC modality, normalization was performed using RunTFIDF. Cells were filtered based on strict QC metrics: nFeature_RNA (500–9,000), percent.mt (< 20%), nCount_ATAC (> 1,500), TSS enrichment (±2‐kb window) (> 2), FRiP (> 0.2), and nucleosome signal (< 1.5). Additionally, potential doublets were identified and removed using scDblFinder (v1.20.2) [88].

To account for biological and technical replicates, Harmony (v1.2.3) was utilized for batch effect correction across samples. Dimensionality reduction was performed via PCA (RNA) and SVD (ATAC). We employed Weighted Nearest Neighbor (WNN) analysis to integrate the two modalities, constructing a multimodal graph to resolve cell states. Cell type annotation was performed by combining Allen Brain Atlas label transfer (using FindTransferAnchors and TransferData) with manual curation based on canonical marker genes. Clusters were identified using the Leiden algorithm on the WNN graph, and cluster‐specific chromatin accessibility was analyzed using MACS2 for peak calling.

### 4.19 Single‐cell Data of Mouse Testis Processing and Quality Control

The analysis of single‐cell RNA‐seq and scATAC‐seq data derived from mouse testis tissue was conducted using the Seurat (v5.3.0) and Signac (v1.14.0) frameworks. Rigorous quality control (QC) procedures were applied independently to each modality. For the scRNA‐seq modality, cells were retained if they met the following criteria: nFeature_RNA between 300 and 9,000, nCount_RNA below 50,000, mitochondrial content less than 25%, and ribosomal content under 10%. In parallel, scATAC‐seq data were filtered based on specific thresholds: nFeature_ATAC > 400, nCount_ATAC < 100,000, TSS enrichment score > 2, blacklist ratio < 0.075, nucleosome signal < 1.5, and a FRiP score > 0.16. To ensure data integrity, potential doublets were identified and removed using scDblFinder (v1.20.2). To account for technical variation, Harmony (v1.2.3) was employed to perform batch effect correction across replicates.

Following QC, scRNA‐seq data were normalized and scaled using the SCTransform method while regressing out mitochondrial content. Dimensionality reduction was performed via Principal Component Analysis (PCA), and the top 50 principal components were utilized for graph‐based clustering (using FindNeighbors and FindClusters). For chromatin accessibility profiling, we executed Latent Semantic Indexing (LSI) dimensionality reduction on the top 50% of variable peaks (components 2–40). To resolve complex cell states in the testis, Weighted Nearest Neighbor (WNN) analysis was utilized to integrate the RNA and ATAC modalities into a unified multimodal graph. Cell type annotations were curated by combining canonical marker gene expression with automated label transfer from the scRNA‐seq dataset. Furthermore, a gene activity matrix was generated using the GeneActivity function, and transcription factor (TF) motif enrichment analysis was performed using chromVAR (v1.30.1) [89] with the JASPAR 2020 database (v0.99.10) to identify differentially active TFs across testicular cell lineages.

### 4.20 Identification of Robust Peak‐Gene Links

To identify biologically relevant regulatory interactions, we restricted the peak‐to‐gene linkage assessment to high‐confidence, cell‐type‐specific markers. Initially, differentially expressed genes (DEGs) were identified using the FindAllMarkers function on the SCT‐normalized expression data, with parameters set to only.pos = TRUE, min.pct = 0.1, and logfc.threshold = 0.25.

The resulting candidate list was refined by excluding poorly annotated transcripts and predicted gene models (e.g., Gm or Rik prefixes) to minimize noise and improve the interpretability of the regulatory network. Furthermore, we calculated a specificity score for each gene, defined as the difference between the fraction of expressing cells inside and outside the target cluster (pct.1 – pct.2). Only genes with a specificity score > 0.1 and a significant p‐value (< 0.05) were retained for downstream analysis. Finally, the LinkPeaks function in Signac (v1.14.0) was employed to calculate the correlation between chromatin accessibility at distal elements and the expression of these curated specific markers, utilizing a distance window of 500 kb from the gene transcription start site.

### 4.21 Identification of Germ Cell Subtypes

To capture the biological heterogeneity within specific germline stages, we performed high‐resolution sub‐clustering analyses on two distinct cell populations: Spermatogonia (SPG) and the Spermatid lineage (comprising both round and elongating spermatids). These subsets were extracted from the main dataset and re‐processed using the Seurat (v5.3.0) and Signac (v1.14.0) pipelines. For each sub‐population, we re‐identified the top 3,000 highly variable genes to drive the dimensionality reduction and ensure the capture of lineage‐specific transcriptomic features.

Clustering was executed using the Louvain algorithm with multilevel refinement (Seurat algorithm = 1 or 2), with the resolution parameter set to 1. The resulting sub‐clusters were annotated based on the expression of canonical marker genes. Subsequently, cluster‐specific differentially expressed genes (DEGs) and peaks were identified using the FindAllMarkers function. To interpret the biological significance of these DEGs, Gene Ontology (GO) enrichment analysis was performed utilizing the clusterProfiler package (v4.14.3) [90], with p‐value adjusted for multiple testing using the Benjamini‐Hochberg method.

### 4.22 Trajectory Inference and Dynamic Regulatory Analysis

To elucidate the developmental hierarchy, we applied monocle3 (v1.3.7) [91] to infer pseudotime trajectories for SPG cells and the spermatid lineage (comprising round and elongating spermatids). The processed Seurat objects were converted into cell_data_set format, and trajectories were constructed using the learn_graph function with default parameters. Lineage‐associated genes exhibiting pseudotemporal dependencies were initially identified via spatial autocorrelation analysis (Moran's I).

To focus on potential epigenetically regulated drivers, we intersected these lineage‐associated genes with the high‐confidence peak‐gene pairs identified through our LinkPeaks analysis. The pseudotemporal expression patterns of these overlapping genes were visualized using heatmaps. We further categorized these genes into distinct co‐expression modules using kNN‐DREMI dynamic clustering, followed by Gene Ontology (GO) enrichment analysis to characterize the biological functions associated with each module.

Complementarily, scVelo (v0.3.3) [92] was employed to resolve transcriptional kinetics specifically for the round and elongating spermatid populations. Spliced and unspliced count matrices were assembled into an AnnData object, filtered, and normalized with the top 2,000 highly variable genes retained. Moments were computed based on the first 30 principal components and 30 neighbors. We recovered the latent transcriptional dynamics and calculated velocity vectors using the “dynamical” mode, restricting the analysis to genes with high goodness‐of‐fit scores to ensure robustness. Finally, the inferred RNA velocities were projected onto the UMAP embedding to visualize developmental directionality.

### 4.23 Identification of DORCs and Regulatory Network Construction

The FigR (v0.1.0) [25] workflow was adopted to determine Domains of Regulatory Chromatin (DORCs) and their upstream regulators. Instead of topic modeling, we utilized LSI‐based nearest neighbor embedding to model the cellular manifold, ensuring consistency with our multimodal integration. We assessed *cis*‐regulatory associations by correlating gene expression with local chromatin accessibility, filtering for statistically significant links with a *pval*Z ≤ 0.05.

Genes exhibiting an exceptionally high density of *cis*‐regulatory interactions (≥ 4 associated peaks) were classified as DORCs, representing key nodes of epigenetic control. Finally, transcription factor (TF) activity was integrated with these DORCs to infer a directed regulatory network, which was subsequently analyzed for network topology and hub detection using igraph (v2.1.4) [93].

### 4.24 CellOracle‐based Regulatory Simulation

To validate the functional roles of key regulators, we performed in silico perturbation analysis utilizing the CellOracle (v0.20.0) framework [94]. The workflow commenced with the identification of co‐accessible chromatin regions via Cicero (v1.3.9), which served as the scaffold for the underlying regulatory landscape.

We then inferred cluster‐specific Gene Regulatory Networks (GRNs) by integrating the refined gene expression data. To ensure network robustness, valid regulatory links were restricted to those meeting a significance level of 0.001, retaining the top 2,000 strongest links per cluster. For the perturbation assay, we simulated a “loss‐of‐function” phenotype by artificially zeroing out the expression of target TFs. Finally, the resulting changes in developmental flow were evaluated by comparing the perturbed transition vectors against the original differentiation trajectory to predict the lineage‐specific impact of each regulator.

### 4.25 CUT&Tag Analysis

Testes were harvested from male C57BL/6J mice at postnatal day 7 (P7), P21, P35, and 10 weeks of age (10 W). Testicular tissue was dissociated into single‐cell suspensions using the two‐step enzymatic digestion described above (Cell dissociation from mouse testis), and the cell suspension was filtered to remove debris. Cells were collected by centrifugation and subjected to CUT&Tag using antibodies against CREM (FineTest, FNab01970) and RFX3 (Proteintech, 14784‐1‐AP). CUT&Tag assays were performed according to the tandard CUT&Tag protocol. Briefly, cells were bound to concanavalin A‐coated magnetic beads, permeabilized, and incubated with primary antibody overnight at 4°C. After secondary antibody incubation, protein A‐Tn5 fusion protein (pA‐tn5) was added, and tagmentation was performed in Mg^2^^+^‐containing buffer at 37°C. Tagmented DNA was extracted, purified, and PCR‐amplified to generate sequencing libraries. One library was generated per time point and per transcription factor; no biological or technical replicates were included.

Raw FASTQ data were trimmed with Trimmomatic (v0.39) to remove adapter sequences. Trimmed reads were mapped to the mouse mm10 genome using Bowtie2 (v2.5.4) with default parameters. Peaks were called using MACS2 (v2.2.9.1) with a p‐value threshold of 0.01. Bigwig files were generated and visualized using Integrative Genomics Viewer (IGV). The reduce function from the GenomicRanges package was used to merge peaks, and overlapping regions between CUT&Tag peaks and other genomic features of interest (e.g. scATAC‐seq peaks) were identified.

## Author Contributions

L.Z. and D.C. conceived the study and developed the DUET‐seq methods; D.C. conducted all DUET‐seq experiments with the assistance of P.Y., Z.L., T.L., M.Z. and M.X.; L.W., C.L., M.Z. and M.X. contributed to beads design and generation. Z.M. and D.C. led the bioinformatics analyses; D.C., Z.M., P.Y. and W.L. prepared the figures; M.X. helped with data processing. D.C., P.Y. and F.Z. contributed to the mouse brain sampling; D.C., P.Y. and F.Z. contributed to the mouse testis sampling; L.Z. and Y.B. acquired the funding; L.Z., Y.B., and A.H. supervised the study; The manuscript was written by D.C.; All authors read and approved the manuscript.

## Funding

This work was supported by the Prevention and Control of Emerging and Major Infectious Diseases‐National Science and Technology Major Project (Grant No. 2025ZD01905600), National Natural Science Foundation of China (Grant No. 82372356 and 82372630), Chongqing Talents Program‐Youth Top‐notch Talents (Grant No. CQYC20220512141 and 20880), CQMU Program for Youth Innovation in Future Medicine (Grant No. W0161 and W0143).

## Conflicts of Interest

The authors declare no conflicts of interests.

## Supporting information




**Supporting File 1**: advs77986‐sup‐0001‐SuppMat.docx.

**Supporting File 2**: advs77986‐sup‐0002‐SuppMat.xlsx.

## Data Availability

The raw sequencing data and processed datasets generated in this study have been deposited in the Gene Expression Omnibus (GEO) under accession number GSE345817 and are publicly available. The following publicly available datasets were utilized for benchmarking and comparative analysis: 10x Multiome and ISSAAC‐seq data from ArrayExpress (E‐MTAB‐11264); sci‐CAR‐seq (GSE117089), SHARE‐seq (GSE140203), SNARE‐seq2 (GSE157660), Paired‐seq (GSE130399), and SUM‐seq (GSE253165) from the Gene Expression Omnibus (GEO); and HT‐scCAT‐seq from GSA (CRA025996). The 10x Multiome mouse brain dataset was downloaded from the 10x Genomics repository. All original code and analysis scripts (including the barcode whitelist and complete oligonucleotide sequence list) used to support the findings of this study have been deposited in a GitHub repository (https://github.com/biochengd/DUET‐seq).
