# Supplementary material for: DUET‐seq: An Open‐Source Droplet Platform for High‐Fidelity Joint Chromatin and Transcriptome Profiling Reveals Temporal Regulatory Decoupling in Single Cells
Source: Adv Sci (Weinh). 2026 Sep 27:e77986. Online ahead of print. doi: 10.1002/advs.77986 (PMC13616282; doi:10.1002/advs.77986)
Supplement: Supplementary file 1 [file ADVS-9999-e77986-s002.docx]

**Supplementary information**

**DUET-seq: An Open-Source Droplet Platform for High-Fidelity Joint Chromatin and Transcriptome Profiling Reveals Temporal Regulatory Decoupling in Single Cells**

Dong Cheng^1, #^, Zijun Meng^2, #^, Lan Wei^3, #^, Pingjing Yang^4, #^, Fanfan Zhang^4^, Zhiyi Luo^4^, Ting Lai^4^, Chunli Li^5^, Mengyao Zhao^4^, Mengqin Xu^4^, Jiaqi Wang^4^, Linjun Li^6^, Huarong Chen^7^, Jin Li^8, *^, Ailong Huang^4, *^, Youquan Bu^1, 9, *^, Liuyang Zhao^4, 10, *^

^1^ Department of Thoracic Surgery, The First Affiliated Hospital of Chongqing Medical University; Department of Biochemistry and Molecular Biology, College of Basic Medical Sciences, Chongqing Medical University, Chongqing 400016, China;

^2^ Chongqing Key Laboratory of Translational Medical Research in Cognitive Development and Learning and Memory Disorders, Ministry of Education Key Laboratory of Child Development and Disorders, National Clinical Research Center for Child Health and Disorders, China International Science and Technology Cooperation Base of Child Development and Critical Disorders, Children’s Hospital of Chongqing Medical University, Chongqing 400015, China

^3^ Chongqing Blood Center, Chongqing 400052, China;

^4^ Key Laboratory of Molecular Biology on Infectious Diseases (Ministry of Education), Chongqing Medical University, Chongqing 400016, China;

^5^ Department of Hepatobiliary Surgery, The Second Affiliated Hospital of Chongqing Medical University, Chongqing 400010, China;

^6^ Department of Cardiothoracic Surgery, The First Affiliated Hospital of Chongqing Medical University, Chongqing 400016, China;

^7^ Department of Anaesthesia and Intensive Care and Peter Hung Pain Research Institute, The Chinese University of Hong Kong, Hong Kong, China;

^8^ Department of Laboratory Medicine, The Affiliated Dazu’s Hospital of Chongqing Medical University, Chongqing 402360, China

^9^ Molecular Medicine and Cancer Research Center, Chongqing Medical University, Chongqing 400016, China;

^10^ Reproductive Medicine Center, the First Affiliated Hospital of Chongqing Medical University, Chongqing 400016, China;

^#^ These authors contributed equally: Dong Cheng, Zijun Meng, Lan Wei, Pingjing Yang

^*^ These authors are co-corresponding authors, and Liuyang Zhao is the lead contact author for this paper.

**Correspondence:** [liuyangzhao@cqmu.edu.cn](mailto:liuyangzhao@cqmu.edu.cn); [buyqcn@cqmu.edu.cn](mailto:buyqcn@cqmu.edu.cn); [ahuang@cqmu.edu.cn](mailto:ahuang@cqmu.edu.cn); [lijin@hospital.cqmu.edu.cn](mailto:lijin@hospital.cqmu.edu.cn);

**Supplementary fig 1. DUET-seq library architecture and bead oligonucleotide design.** The complete sequence architecture of the dual-linker barcoded hydrogel beads is shown, including barcode, UMI, and terminal capture/priming modules for RNA and ATAC. A stepwise schematic illustrates the DUET-seq workflow and the corresponding oligonucleotide sequences and library structures for both RNA and ATAC modalities across key stages, including transposition, droplet co-encapsulation, one-step RT–PCR, co-amplification, and final indexing.

**Supplementary fig 2. DUET-seq microfluidics and library quality control.**

**a.** Schematic of the microfluidic chip used for hydrogel bead generation. Inlets correspond to carrier oil and PEG–primer mix, with droplets collected from the outlet. Channel depth is 50 µm. Passive inlet filters are included to reduce clogging from dust and debris.

**b.** Schematic of the microfluidic chip used for single-nucleus encapsulation. Four inlets deliver carrier oil, RT–PCR master mix, barcoded hydrogel beads, and nuclei. Channel depth is 80 µm. Passive inlet filters are included to reduce clogging from dust and debris.

**c.** Diagram and representative snapshot showing co-encapsulation of nuclei, a single barcoded hydrogel bead and RT-PCR buffer into droplets. Arrows indicate flow direction.

**d.** Photograph of the emulsion after droplet RT–PCR, showing phase separation into an upper mineral oil phase, a middle emulsion layer, and a lower carrier oil phase before emulsion breaking.

**e.** Representative brightfield (left) and fluorescence (right) micrographs show monodisperse droplets (diameter ~120 µm; volume ~0.9 nL) and efficient bead encapsulation.

**f.** Bioanalyzer electropherograms of representative libraries. Left, RNA (cDNA) library after PCR amplification. Right, ATAC library showing nucleosomal periodicity.

**g.** Unique RNA UMIs aligning to the human or mouse genome in a species-mixing experiment using HEK293T and NIH/3T3 cells.

**h.** Enrichment of ATAC-seq reads around transcription start sites (TSSs) (left) and the insert size distribution of ATAC-seq fragments (right).

**i.** Genomic annotation of mapped ATAC fragments (left) and transcripts (right) in HEK293T and NIH/3T3 cells.

**Supplementary fig 3. Systematic optimization of joint chemistry for the DUET-seq platform.**

**a.** Schematic of the tiered optimization strategy.

**b.** RNA module optimization—buffer and additives. Box plots showing how different concentrations of detergents and additives (CA-630, MgCl₂, NaCl, Tris-HCl, dCTP, dNTP, PEG8000, betaine, and DTT) affect transcript capture sensitivity. Statistical significance was assessed by two-sided Wilcoxon rank-sum test; *P < 0.05, **P < 0.01, ***P < 0.001.

**c.** RNA module optimization—enzyme concentrations. Box plots depicting the effect of varying Template Switching Oligo (TSO) and Maxima H-Minus Reverse Transcriptase (RTase) concentrations on transcript capture sensitivity. Statistical significance was assessed by two-sided Wilcoxon rank-sum test; *P < 0.05, **P < 0.01, ***P < 0.001.

**d.** RNA module optimization—workflow parameters. Box plots evaluating the effects of RNA separation conditions (streptavidin bead incubation volume) and microfluidic droplet size (90–130 µm) on RNA data quality. Statistical significance was assessed by two-sided Wilcoxon rank-sum test; *P < 0.05, **P < 0.01, ***P < 0.001.

**e.** ATAC module optimization—buffer and additives. Violin plots showing the Fraction of Reads in Peaks (FRiP) scores under different concentrations of MgCl₂, PEG8000, DTT, and dNTPs. Statistical significance was assessed by two-sided Wilcoxon rank-sum test; *P < 0.05, **P < 0.01, ***P < 0.001.

**f.** ATAC module optimization—wash times. Violin plots showing the impact of different wash cycles (no wash, 1×, or 2×) following bulk transposition on the final FRiP score. Statistical significance was assessed by two-sided Wilcoxon rank-sum test; *P < 0.05, **P < 0.01, ***P < 0.001.

**g.** ATAC module optimization—linear PCR cycles. Violin plots showing how varying the number of linear PCR cycles (5, 10, or 15) during the intra-droplet reaction affects chromatin accessibility signal quality. Statistical significance was assessed by two-sided Wilcoxon rank-sum test; *P < 0.05, **P < 0.01, ***P < 0.001.

**h.** Cross-modality contamination rates in both directions. Chromatin-derived reads in RNA libraries (left) and RNA-derived reads in ATAC libraries (right), as a percentage of all modality-assigned reads.

**i.** Frequency of TSO motifs (left) and exon–exon junction-spanning fragments (right) in ATAC libraries from DUET-seq (blue) and 10x Multiome (orange).

**j.** Additive effects of decoy DNA and blocking oligonucleotide on cross-modality contamination.

**Supplementary fig 4. Quality control and cross-platform comparison of DUET-seq data in mouse brain.**

**a.** Violin plots comparing key quality control metrics between DUET-seq and 10x Multiome datasets, including mitochondrial read fraction (percent.mt), total unique ATAC fragments (nCount_ATAC), TSS enrichment score, and FRiP (Fraction of Reads in Peaks).

**b.** Pseudo-bulk correlation analysis (Spearman’s ρ) for RNA (left) and ATAC (right) profiles between DUET-seq and 10x Multiome, and the corresponding two-sided p-value are shown on each panel.

**c.** Cell-type specificity visualization. Left: normalized expression of top differentially expressed genes (DEGs) per cell type. Right: cell-type-specific TF motif accessibility scores computed by chromVAR.

**d-f.** UMAP embeddings of integrated mouse brain datasets (DUET-seq and 10x Multiome) using (d) RNA-based integration, (e) ATAC-based integration, and (f) WNN (weighted nearest neighbor) integration. Cells are colored by platform to assess alignment and batch effects.

**g.** Bar plot comparing the proportions of major cell types recovered by DUET-seq and 10x Multiome based on integrated annotations.

**h.** Histogram showing the distribution of significant peak-to-gene (P2G) linkages per gene (p < 0.05; within ±50 kb of the transcription start site). Genes with exceptionally high numbers of linkages, including DORC-associated genes, are highlighted.

**i.** Representative DORCs for each cluster. Activity values are min–max normalized within each DORC.

**Supplementary fig 5. Data quality assessment for DUET-seq mouse brain RNA profiles.**

UMAP projection of the DUET-seq RNA modality. Cells are colored by normalized expression of representative cell-type marker genes (panels 1–29). The bottom-right panel shows the same UMAP colored by biological replicate (Rep1 and Rep2), indicating robust integration and minimal batch effect.

**Supplementary fig 6. Quality control of the DUET-seq mouse testis dataset (related to Fig. 4).**

**a-b.** Boxplots summarizing key QC metrics across all testis libraries (n = 18; six time points with three replicates each). (a) RNA metrics: number of detected genes (left) and number of UMIs (right). (b) ATAC metrics: unique fragment counts (left) and FRiP scores (right).

**c.** ATAC quality metrics: enrichment of reads around transcription start sites (TSS; left) and fragment size distribution (right).

**d.** Heatmaps of Spearman correlation coefficients for pseudo-bulk profiles across developmental stages, for RNA (left) and ATAC (right), showing high within-stage correlation and stage-associated progression patterns. All pairwise correlations were significant (two-sided P < 0.001). Color intensity represents the Spearman correlation coefficient.

**e.** UMAP embeddings of all testis nuclei colored by developmental stage (P7–10W) for RNA (top) and ATAC (bottom), demonstrating stage-specific structure with minimal batch effects.

**f.** Bar plot showing the cell type composition across all 18 libraries.

**g.** FRiP after depth-controlled downsampling. Libraries were downsampled to matched unique fragment depths and FRiP was recalculated; the developmental decline from P7 to 10W is preserved, indicating that it is not attributable to differences in fragment depth or library complexity alone.

**h.** FRiP resolved by germ-cell state within each developmental stage. Round and elongating spermatids show consistently lower FRiP than spermatogonia and spermatocytes of the same age.

**i.** Summary of the analysis in (h) across all six time points (P7–10W), showing that the spermatid-associated FRiP reduction is reproduced at every stage at which spermatids are present.

**j.** The same fragment-level FRiP calculation applied to an independent, publicly available mouse testis Multiome dataset (Whelan et al.³²), in which spermatids likewise show lower FRiP than spermatogonia and spermatocytes. Together, (g–j) indicate that the FRiP decline reflects a germ-cell-state-associated feature rather than a DUET-seq-specific technical artifact.

**Supplementary fig 7. Validation of cell-type annotation and integration in the mouse testis profile.**

**a.** UMAP projection of the RNA modality, with cells colored by normalized expression of 18 marker genes used to confirm the identity of major testis cell clusters.

**b.** Dot plots showing average gene expression (RNA, left) and gene activity (ATAC, right) for key markers across developmental stages. Dot size represents the percentage of expressing cells, and color intensity indicates average scaled expression or activity.

**c.** Distribution of Sertoli cell subclusters across developmental stages (P7–10W). Top: RNA-defined Sertoli subclusters; bottom: ATAC-defined Sertoli subclusters.

**d.** Sankey diagram showing correspondence between RNA clusters (R1–R2) and ATAC clusters (A1–A4) within Sertoli cells.

**e.** Gene Ontology (GO) enrichment analysis for RNA-defined Sertoli clusters.

**f.** Heatmap of aggregated ATAC signal across the top 200 cluster-specific peaks for Sertoli ATAC subclusters (A1–A4), highlighting subcluster-specific accessibility patterns.

**Supplementary fig 8. Characterization of spermatogonial heterogeneity and the self-renewal regulatory network.**

**a.** Scatter plots showing the top differentially expressed genes between Early (P7–P14) and Late (P21–10W) spermatogonial populations. Some of the top-ranked genes have been labeled.

**b.** UMAP feature plots of stage-specific marker genes used to annotate spermatogonial subpopulations.

**c.** Heatmap of putative target genes associated with the two TF modules identified in Fig. 5i.

**d.** Gene Ontology (GO) enrichment analysis for target genes of the Self-renewal (Module 1) and Differentiation (Module 2) TF networks.

**e.** Histogram showing the distribution of significant peak-to-gene (P2G) linkages per gene (p < 0.05; within ±50 kb of the transcription start site). Genes with exceptionally high numbers of linkages, including key DORC-associated genes, are highlighted.

**f.** Scatter plot of *in silico* perturbation scores. TFs are ranked by their predicted influence on the SSC state (x-axis) versus the meiotic state (y-axis) based on in silico perturbation analysis. Crem, Nr2c2, and Atf1 show high predicted influence on differentiation-associated states, whereas Erg is associated with SSC-related states.

**g.** Visualization of the self-renewal gene regulatory network (GRN), highlighting Erg and its predicted regulatory associations with chromatin domains linked to stemness and niche adhesion.

**Supplementary fig 9. Characterization of spermatid substages and the Jund-centered regulatory network.**

**a.** Dot plots comparing gene expression (RNA; left) and gene activity inferred from chromatin accessibility (ATAC; right) for canonical spermiogenesis markers across spermatid substages.

**b.** Histogram showing the distribution of significant peak-to-gene (P2G) linkages per gene (p < 0.05; within ±50 kb of the transcription start site). Genes with exceptionally high numbers of linkages, including key DORC-associated genes, are highlighted.

**c.** FigR-derived TF–DORC association scores highlighting candidate TFs associated with spermiogenesis progression; scatter plot (right) shows top-ranked TFs.

**d.** Multimodal characterization of the master regulator Jund, showing its enriched DNA-binding motif (top), gene expression UMAP (middle), and inferred TF activity UMAP (bottom), consistent with its stage-specific activation pattern.

**e.** Visualization of the RS_Early–specific gene regulatory network (GRN), highlighting key regulatory interactions active during early round spermatid development.

 **Supplementary Fig. 10. Orthogonal validation of CREM and RFX3 occupancy at DUET-seq-predicted regulatory elements by bulk CUT&Tag.**

**a.** Heatmaps and aggregate profiles showing CREM and RFX3 CUT&Tag signals centered on spermatogonial progenitor cell (SPG)-linked ATAC peaks identified by DUET-seq. CUT&Tag enrichment was assessed across mouse testis developmental stages (P7, P21, P35, and 10 weeks [10W]). Both CREM and RFX3 showed enrichment at DUET-seq-nominated regulatory elements, with the strongest occupancy observed at P7.

**b.** Genome browser tracks illustrating CREM and RFX3 CUT&Tag signals at the representative priming locus Pde4b. ATAC-seq accessibility tracks from matched developmental stages are shown below for comparison. Highlighted regions indicate regulatory elements identified by DUET-seq as associated with spermatogonial lineage genes. CREM and RFX3 occupancy was detected at accessible regulatory regions before the induction of linked gene expression, consistent with a potential role in transcriptional priming.

**Supplementary Table**

**Supplementary Table 1.** DUET-seq Primer Sequences.

**Supplementary Table 2.** DUET-seq barcode Sequences.

**Supplementary Table 3.** Detailed cost and time estimates for DUET-seq.

**Supplementary Table 4.** Throughput, scalability and performance of DUET-seq relative to published single-cell methods for joint profiling of chromatin accessibility and gene expression.

**Supplementary Table 5.** Mouse brain quality control.

**Supplementary Table 6.** Cell type-specific transcriptomic and epigenomic signatures in the adult mouse brain.

**Supplementary Table 7.** 14255 significantly linked CRE-gene pairs in mouse brain.

**Supplementary Table 8.** Statistics of Nuclei from Mouse Testis.

**Supplementary Table 9.** 15,016 significantly linked CRE-gene pairs in mouse testis.

**Supplementary Table 10.** Top five putative regulators in each major cell type.

**Supplementary Table 11.** Top 20 GO biological processes derived from the cluster analysis of sertoli cells.

**Supplementary Table 12.** Top 20 DEGs of the early and later stage in spermatogonia.

**Supplementary Table 13.** Top 20 DEGs for each developmental stage in spermatogonia.

**Supplementary Table 14.** DORCs and their associated TFs from SPG_SSC to SPG_Meiotic trajectory

**Supplementary Table 15.** In silico TF perturbation in the SPG_SSC and SPG_Meiotic.

**Supplementary Table 16.** Crem targets predicted in SPG_SSC to SPG_Meiotic lineage.

**Supplementary Table 17.** Top 20 DEGs for each developmental stage in RS/ES.

**Supplementary Table 18.** DORCs and their associated TFs from RS_Early to ES_Early trajectory.

**Supplementary Table 19.** In silico TF perturbation in the RS_Early to ES_Early.

**Supplementary Table 20.** Jund targets predicted in RS_Early to ES_Early lineage.

**Supplementary Protocol**

**Reagents**

•	PBS (Thermo Fisher Scientific, Cat#10010-23)

•	Tris-HCl (Sigma, Cat# 10812846001)

•	MgCl_2_ (Sigma, Cat#63069)

•	NaCl (Sigma, Cat#S7653)

•	EDTA (Invitrogen, Cat#AM9261)

•	IGEPAL CA-630 (Sigma, Cat#I8896)

•	Tween-20 (Sigma, Cat# P2287-100ML)

•	Digitonin (Promega, Cat# G9441)

•	dCTP solution (Yeasen, Cat#10119ES96)

•	dNTP mix (Yeasen, Cat#10125ES90)

•	Droplet Generation Oil (BioRad, Cat#1864112)

•	TruePrep Tagment Enzyme (Vazyme, Cat#S601)

•	cOmplete EDTA-free Protease Inhibitor Cocktail (Roche, Cat#05056489001)

•	Murine RNase Inhibitor (Yeasen, Cat#10603ES60)

•	Recombinant RNase Inhibitor (Takara, Cat# 2313B)

•	RiboLock RNase Inhibitor (Invitrogen, Cat#EO0384)

•	KAPA HiFi HotStart DNA Polymerase (Roche, Cat#KK2502)

•	Maxima H Minus Reverse Transcriptase (Thermo Fisher Scientific, Cat#EP0753)

•	VAHTS DNA Clean Beads (Vazyme, Cat#N411-03)

•	Qubit dsDNA HS Assay Kit (Thermo Fisher Scientific, Cat#Q32854)

•	GoldBand 100 bp plus DNA Ladder (Yeasen, Cat#10516ES80)

•	Oligonucleotides and primers (Sangon, see Supplementary Table1)

**Equipments**

•	Benchtop centrifuge (Eppendorf)

•	T100 Thermal Cycler (Bio-Rad)

•	ThermoMixer (Eppendorf)

•	Swing bucket centrifuge (ThermoFisher)

•	Qubit Fluorometric Quantification (ThermoFisher)

•	Magnetic stands (96-well plate stand, PCR-tube stand)

•	Fragment analyzer/Tapestation (Agilent)

•	Micropipetts (0.5-20 µL, 10-200 µL, 100-1000 µL)

•	DNA LoBind tubes (1.5 mL, Eppendorf #L200934G)

•	Maximum recovery tubes (1.5 mL, Axygen, MCT150LC)

**Reagent setup**

**Omni-ATAC RSB**

Mix 500 µL 1 M Tris-HCl (pH 7.5), 100 µL 5 M NaCl, 150 µL 1 M MgCl2, and 49.25 mL Nuclease-free Water for 50 mL omni-ATAC RSB. Sterilize through 0.22 μm filter, stored at 4°C for up to 2 months.

**4× THS TD buffer**

Mix 132 µL 1 M Tris-HCl (pH8.0), 52.8 µL 5 M Potassium acetate, 40 µL 1 M Magnesium acetate, 640 µL N, N-Dimethylformamide (DMF) and 135.2 µL Nuclease-free Water for 1 mL 4× THS TD buffer. Store at -20°C for up to 2 months.

**2****× Stop Buffer**

Mix 500 µL 1× PBS, 40 μl 0.5M EDTA (pH8.0), 66 μl 30% BSA and 394 µL Nuclease-free Water for 1 mL 2× Stop Buffer. Make fresh.

**1× DPBS-0.5%BSA-RI**

Mix 166.7 µL 30% (w/v) BSA, 50 µL Murine RNase Inhibitor (40 U/µL), and 10 mL 1× DPBS. The buffer can be stored at -20°C for up to 2 weeks.

**Digitonin**

The recommended digitonin from Promega is supplied at a 2% (wt/vol) concentration in DMSO. Dilute this digitonin 1:1 with water to make a 1% (wt/vol) (100×) stock solution. This DMSO–water mixture will no longer freeze at −20 °C. Store at −20 °C for up to 6 months.

**50% (w/w) PEG8000**

Mix 5 g PEG8000 and 5 mL Nuclease-free Water, sterilize through 0.22 μm filter, aliquot and store at -20°C for up to 2 months.

**10% (vol/vol) Tween-20**

Mix 5 mL Tween-20 and 5 mL Nuclease-free Water. Sterilize through 0.22 μm filter, aliquot and store at 4°C for ≤1 year.

**10% (vol/vol) IGEPAL CA-630**

Mix 5 mL IGEPAL CA-630 and 5 mL Nuclease-free Water. Sterilize through 0.22 μm filter, aliquot and store at 4°C for ≤1 year.

**10% BSA（100mg/mL）**

Place 5 g of BSA in a 50 mL conical tube and add 20°C–25°C nuclease-free water to the 50-mL tube. Mix (e.g., rocking, rotation) at 20°C–25°C until dissolved and adjust the final volume to 50 mL with 20°C–25°C tube. Sterile filter into a fresh 50-mL conical with a 50-mL disposable syringe and attached 0.45-µm sterile filter. Store 10 mL aliquots at −20°C for up to 1 year, keep a working aliquot at 4°C for up to 3 months.

**Procedures**

**Assemble Tn5 transposome complex**

1. Dissolve ME_s5, ME_s7, and ME_bottom oligos in 0.1× TE buffer to a final concentration of 10 µM.
2. Mix 10 µL ME_s5 oligo (10 μM) with 10 µL ME_bottom oligo (10 μM) in a PCR tube, and anneal in a thermocycler as follows: 98 °C for 3 min, and slowly cool down to 16 °C with a temperature ramp of -0.1 °C/s, to generate s5_adapter (5 μM).
3. Similarly, mix 10 μl ME_s7 oligo (10 μM) with 10 µL ME_bottom oligo (10 μM) in a PCR tube and anneal to form s7_adapter (5 μM).
4. Mix the combined adapters in equal volumes, label the resulting mixture as adapter mix.
5. To assemble Tn5 transposome, add 7 µL adapter mix, 4 µL TruePrep Tagment Enzyme (Vazyme, 500 ng/µL), and 39 µL coupling buffer to a PCR tube. Mix thoroughly by gently pipetting up and down 20 times.
6. Incubate at 30 °C for 1 h, then store at -20 °C for up to one months.

**Prepare nuclei from cell lines Timing 1 h**

1. If using fresh cells, perform 1-2 washes with 1× PBS + 0.5% BSA + RI and determine cell count, before proceeding to next step. Nuclei may be isolated from 100,000-1,000,000 cells using this protocol.
2. Add 100,000 -1,000,000 cells to a 1.5 mL microcentrifuge tube. Centrifuge at 500*g* for 3-5 min at 4 °C.
3. Remove all the supernatant without disrupting the cell pellet.
4. Resuspend cells in 100µL chilled ATAC Lysis Buffer. Pipette gently mix 10x. ATAC-seq Lysis Buffer should be made fresh each time and mixed thoroughly prior to use.

|  | **Stock** | **Final** | **Volume** |
| --- | --- | --- | --- |
| Omni-ATAC RSB | - | - | 93 µL |
| 10% Tween-20 | 10% | 0.1% | 1 µL |
| 10% IGEPAL CA-630 | 10% | 0.1% | 1 µL |
| 1% Digitonin | 1% | 0.01% | 1 µL |
| Murine RNase Inhibitor | 40 U/µL | 1 U/µL | 2.5 µL |
| Recombinant RNase Inhibitor | 40 U/µL | 1 U/µL | 2.5 µL |
| Total |  |  | 100 µL |

5. Incubate for 3 min on ice. If lysing multiple samples, make sure that all samples are lysed for the same total amount of time by proceeding to next steps. Assess lysis efficacy via the microscopy.
6. Add 1 mL chilled ATAC Wash Buffer to the lysed cells, invert the tube 5x to mix. ATAC Wash Buffer should be made fresh each time and mixed thoroughly prior to use.

|  | **Stock** | **Final** | **Volume** |
| --- | --- | --- | --- |
| Omni-ATAC RSB | - | - | 990 µL |
| 10% Tween-20 | 10% | 0.1% | 10 µL |
| Total |  |  | 1 mL |

7. Centrifuge at 800*g* for 5 min at 4°C.
8. Remove the supernatant without disrupting the nuclei pellet.

**Chromatin tagmentation**

1) In each tagmentation reaction, there are 100,000 nuclei. Prepare 100 µL chromatin tagmentation reaction mixture for each sample：

|  | **Stock** | **Final** | **Volume** |
| --- | --- | --- | --- |
| 4×THS TD buffer | 4× | 1× | 25 µL |
| 1% Digitonin | 1% | 0.01% | 1 µL |
| Murine RNase Inhibitor | 40 U/µL | 1 U/µL | 2.5 µL |
| Recombinant RNase Inhibitor | 40 U/µL | 1 U/µL | 2.5 µL |
| RiboLock RNase Inhibitor | 40 U/µL | 0.5 U/µL | 1.25 µL |
| Tn5 transposome complex | 40 ng/µL | 1 ng/µL | 2.5 µL |
| Nuclease-free water | - | - | 65.25 µL |
| Total |  |  | 100 µL |

2) Resuspend each pellet in 100 µL tagmentation reaction mixture by gently pipetting up and down 10x. Mix gently and avoid bubbles.
3) Incubate at 30°C, 800 rpm for 30 min.
4) Add 100 µL 2× Stop buffer to each sample and gently pipette up and down 5 times to mix. Incubate for 5 min on ice.

2× Stop buffer:

|  | **Stock** | **Final** | **Volume** |
| --- | --- | --- | --- |
| 1×PBS | - | - | 50 µL |
| EDTA, pH=8.0 | 0.5 M | 20 mM | 4 µL |
| BSA | 10% | 2% | 19.8 µL |
| Nuclease-free water | - | - | 26.2 µL |
| Total |  |  | 100 µL |

5) Centrifuge at 800*g* for 5 min at 4 °C. Remove the supernatants and wash the pellet twice with 500 µL ice-cold 0.5× DPBS + 0.5% BSA + RI (0.2 U/µL). The nuclei pellet may be invisible, be careful not to disrupt the nuclei at the bottom of the tube when aspirating the supernatant. It is okay to have 2-3 µL buffer leftover at this step.
6) Determine the nuclei concentration using a hemocytometer.
7) Resuspend each pellet (final concentration at 1000 nuclei/µL) in ice-cold 1×DPBS + 0.5% BSA + RI (2 U/µL). Keep the suspension on ice until proceeding to the microfluidic droplet encapsulation

**The formation of droplets**

1. Wash the beads by resuspending them in bead resuspend buffer, centrifuging at 1000*g* for 1 minute, taking the supernatant and resuspending them again in bead resuspend buffer. Perform this step a total of three times.

|  | **Stock** | **Final in drop** | **Volume** |
| --- | --- | --- | --- |
| Tris-HCl pH 8.0 | 1 M | 25 mM | 1 |
| NaCl | 1 M | 30 mM | 1.2 |
| MgCl2 | 100 mM | 4 mM | 2.4 |
| IGEPAL CA-630 | 10% | 0.20% | 0.8 |
| Nuclease-free Water | - | - | 34.6 |
| Total |  |  | 40 µL |

Store at -20°C for up to 6 months.

2. After the last wash, take as much supernatant as possible without aspirating any beads. Resuspending beads in 40 µL bead resuspend buffer and keep the beads on ice until you need them.
3. Prepare 120 µL RT-PCR buffer for each sample, slowly pipette up and down 10~20 times to mix.

|  | **Stock** | **Final in drop** | **Volume** |
| --- | --- | --- | --- |
| Tris-HCl pH 8.0 | 1 M | 25 mM | 4 µL |
| NaCl | 1 M | 30 mM | 4.8 µL |
| MgCl2 | 100 mM | 4 mM | 5.6 µL |
| IGEPAL CA-630 | 10% | 0.20% | 3.2 µL |
| PEG8000 | 50% | 1.75% | 7 µL |
| DTT | 100 mM | 8 mM | 16 µL |
| Betaine | 10 M | 1 M | 20 µL |
| dCTP | 100 mM | 3.5 mM | 7 µL |
| dNTP | 25 mM each | 0.5 mM | 4 µL |
| Blocking oligo | 100 µM | 0.5 µM | 1 µL |
| Decoy DNA | 100 µM | 1 µM | 2 µL |
| TSO | 100 µM | 1 µM | 2 µL |
| Murine RNase Inhibitor | 40 U/µL | 1 U/µL | 4 µL |
| Recombinant RNase Inhibitor | 40 U/µL | 0.5 U/µL | 2 µL |
| RiboLock RNase Inhibitor | 40 U/µL | 0.5 U/µL | 2 µL |
| Maxima H minus RTase | 200 U/µL | 4 U/µL | 8 µL |
| KAPA HiFi HotStart DNA Polymerase | 1 U/µL | 0.03 U/µL | 6 µL |
| Nuclease-free Water | - | - | 21.4 µL |
| Total |  |  | 120 µL |

4. Before starting, ensure the chip outlet is connected to waste and the hydrogel beads are properly loaded into the inlet tip. Simultaneously, confirm that the microfluidic pumps, microscope, and thermal cycler are calibrated and ready for the subsequent RT-PCR.
5. Once all four flows have reached the flow focusing point, increase the flow rates: Oil: 18 µL/min; Lysis buffer: 9 µL/min; Cells: 3 µL/min; Beads: 3 µL/min.

**RT-PCR reaction**

1. Carefully transfer 100 µL of the emulsion into each 200 µL PCR tube. Use a syringe to aspirate and remove any excess droplet generation oil from the bottom of the tube, then overlay with a layer of mineral oil to prevent evaporation during incubation.
2. Incubated in a thermocycler using the following program:

| **Program** | **Temperature** | **Duration time** | **No. of cycles** |
| --- | --- | --- | --- |
| RT | 42 °C | 90 min | 1x |
| Gap fill-in | 72 °C | 5 min | 1x |
| PCR | 95 °C | 3 min | 1x |
|  | 98 °C | 20 s | 10x |
|  | 60 °C | 30 s |  |
|  | 72 °C | 1 min |  |
|  | 72 °C | 5 min | 1x |
|  | 12 °C | hold | 1x |

3. Following RT-PCR, the droplets were broken by adding 5 µL (approximately 10% v/v) of 1H,1H,2H,2H-perfluorooctanol (PFO).
4. Remove and discard the oil phase without losing any of the aqueous phase (intermediate phase). Centrifuge at 300*g* at room temperature for 30 s if necessary. It's ok if some oil remains. Aqueous phase appears as a milky (semitransparent) phase.
5. Clean sample with 1.8× VAHTS DNA Clean Beads, elute library to 70 µL 0.1× TE.

**cDNA and** **ATAC DNA co-amplification**

1) Set up one PCR mixture to amplify both cDNA and ATAC DNA:

|  | **Stock** | **Final** | **Volume** |
| --- | --- | --- | --- |
| 5× KAPA HiFi Buffer | 5× | 1× | 20 µL |
| dNTP Mix | 10 mM each | 0.3 mM each | 3 µL |
| TruSeq-R2 primer | 10 µM | 0.4 µM | 4 µL |
| TSO primer | 10 µM | 0.2 µM | 2 µL |
| s5 primer | 10 µM | 0.2 µM | 2 µL |
| KAPA HiFi HotStart DNA Polymerase | 1 U/µL | 0.02 U/µL | 2 µL |
| Purified RT-PCR product | - | - | 67 µL |
| Total |  |  | 100 µL |

2) Incubated in a thermocycler using the following program:

| **Program** | **Temperature** | **Duration time** | **No. of cycles** |
| --- | --- | --- | --- |
| Initial denaturation | 98 °C | 3 min | 1x |
| Denaturation | 98 °C | 20 sec | 7x |
| Annealing | 60 °C | 30 sec |  |
| Elongation | 72 °C | 4 min |  |
| Final elongation | 72 °C | 5 min | 1x |
| Hold | 12 °C | hold | 1x |

3) Splitting PCR products into two equal parts (50 µL for RNA and 50 µL for ATAC, separately).

**ATAC library preparation**

1) Purify ATAC (50 µL) with 70 µL VAHTS DNA Clean Beads (1.4×), and elute in 40 µL nuclease-free water. The ATAC DNA products can be stored at −20 °C.
2) Prepare the second ATAC PCR mixture following the table below.

|  | **Stock** | **Final** | **Volume** |
| --- | --- | --- | --- |
| 5× KAPA HiFi Buffer | 5× | 1× | 10 µL |
| dNTP Mix | 10 mM each | 0.3 mM each | 1.5 µL |
| Nextera i5 index primer | 10 µM | 0.2 µM | 1 µL |
| TruSeq i7 index primer | 10 µM | 0.2 µM | 1 µL |
| KAPA HiFi HotStart DNA Polymerase | 1 U/µL | 0.02 U/µL | 1 µL |
| ATAC DNA Template | - | - | 35.5 µL |
| Total |  |  | 50 µL |

3) Incubated in a thermocycler using the following program:

| **Program** | **Temperature** | **Duration time** | **No. of cycles** |
| --- | --- | --- | --- |
| Initial denaturation | 98 °C | 3 min | 1x |
| Denaturation | 98 °C | 20 sec | 8x |
| Annealing | 60 °C | 30 sec |  |
| Elongation | 72 °C | 1 min |  |
| Final elongation | 72 °C | 5 min | 1x |
| Hold | 12 °C | hold | 1x |

4) Clean sample with 50 µL VAHTS DNA Clean Beads (0.55× - 1.0×), elute library to 25 µL 0.1× TE.
5) Aliquot 2 µL ATAC PCR product for 2% gel electrophoresis.
6) Aliquot 1 µL sample for quantifying cDNA concentration by Qubit.

**Second cDNA PCR**

1) Purify RNA (50 µL) with 35 µL VAHTS DNA Clean Beads (0.7×), and elute in 50 µL 0.1× TE.
2) Prepare the second cDNA PCR mixture following the table below.

|  | **Stock** | **Final** | **Volume** |
| --- | --- | --- | --- |
| 5× KAPA HiFi Buffer | 5× | 1× | 10 µL |
| dNTP Mix | 10 mM each | 0.3 mM each | 1.5 µL |
| TruSeq-R2 primer | 10 µM | 0.2 µM | 1 µL |
| TSO primer | 10 µM | 0.2 µM | 1 µL |
| KAPA HiFi HotStart DNA Polymerase | 1 U/µL | 0.02 U/µL | 1 µL |
| cDNA Template | - | - | 35.5 µL |
| Total |  |  | 50 µL |

3) Incubated in a thermocycler using the following program:

| **Program** | **Temperature** | **Duration time** | **No. of cycles** |
| --- | --- | --- | --- |
| Initial denaturation | 95 °C | 3 min | 1x |
| Denaturation | 98 °C | 20 sec | 7x |
| Annealing | 62 °C | 30 sec |  |
| Elongation | 72 °C | 4 min |  |
| Final elongation | 72 °C | 5 min | 1x |
| Hold | 12 °C | hold | 1x |

4) Purify cDNA with 30 µL VAHTS DNA Clean Beads (0.6×), and elute in 22 µL 0.1× TE buffer. Store the libraries at -20°C.
5) Aliquot 1 µL sample for quantifying cDNA concentration by Qubit.
6) Aliquot 2 µL PCR product for 2% gel electrophoresis.

**RNA library preparation**

1. Quantify cDNA concentration by Qubit. Ideally, need 6 ng cDNA.

Prepare 4×TD buffer (for RNA library):

|  | **Stock** | **Final** | **Volume** |
| --- | --- | --- | --- |
| Tris-HCl (pH 7.5) | 1 M | 40 mM | 40 µL |
| MgCl_2_ | 100 mM | 20 mM | 200 µL |
| Dimethylformamide | 100% | 20% | 200 µL |
| Nuclease-free water | ­- | ­- | 560 µL |

Store at -20°C for up to 6 months.

2. Prepare tagmentation reaction mix：

|  | **Stock** | **Final** | **Volume** |
| --- | --- | --- | --- |
| 4×TD buffer | 4× | 1× | 2.5 µL |
| Tn5-s5/s5 transposome complex | 20 ng/µL | 0.8 ng/µL | 0.4 µL |
| cDNA | - | 6 ng total | - |
| Nuclease-free water | ­- | ­- | - |
| Total |  |  | 10 µL |

3. Incubation in a thermocycler at 55 °C for 10 min.
4. To stop the tagmentation reaction and strip off the Tn5 enzyme, add 2.5 µL of 0.2% SDS to each well. Incubation at room temperature for 5 min.
5. Add 37.5 µL of post-tagmentation PCR mix (below) with 12.5 µL sample.

|  | **Stock** | **Final** | **Volume** |
| --- | --- | --- | --- |
| 5× KAPA HiFi Buffer | 5× | 1× | 10 µL |
| dNTP Mix | 10 mM each | 0.3 mM each | 1.5 µL |
| Nextera i5 index primer | 10 µM | 0.3 µM | 1.5 µL |
| TruSeq i7 index primer | ­10 µM | 0.3 µM | 1.5 µL |
| Tween-20 | 1% | 0.025% | 1.25 µL |
| KAPA HiFi HotStart DNA Polymerase | 1 U/µL | 0.02 U/µL | 1 µL |
| Nuclease Free Water | - | - | 20.75 µL |
| Total |  |  | 37.5 µL |

6. Incubated in a thermocycler using the following program:

| **Program** | **Temperature** | **Duration time** | **No. of cycles** |
| --- | --- | --- | --- |
| Gap-filling | 72 °C | 5 min | 1x |
| Initial denaturation | 98 °C | 3 min | 1x |
| Denaturation | 98 °C | 20 sec | 7x |
| Annealing | 62 °C | 30 sec |  |
| Elongation | 72 °C | 1 min |  |
| Final elongation | 72 °C | 5 min | 1x |
| Hold | 12 °C | hold | 1x |

7. Purify each sample with VAHTS DNA Clean Beads (0.6× - 0.8×), elute library to 25 µL 0.1× TE. Store the libraries at -20°C until quantification.
8. Aliquot 2 µL PCR product for 2% gel electrophoresis.
9. Aliquot 1 µL product for quantifying cDNA concentration by Qubit.

**Library sequencing**

1. DNA and RNA libraries are sequenced in separated runs on the GeneMind SURFSeq 5000 instrument.
2. For DNA libraries, 100 cycles kit (GeneMind #S000365) is used, with cycle number set as 54+8+24+54 (Read1 54 cycles; Index1 8 cycles; Index2 24 cycles; Read2 54 cycles). Index2 read contains cellular barcodes information.
3. For RNA library, 300 cycles kit (GeneMind #S000369) is used, with cycle number set as 150+8+8+150 (Read1 150 cycles; Index1 8 cycles; Index2 8 cycles; Read2 150 cycles). Read2 read contains UMIs and cellular barcodes information.
